# Nucleation and Crystallization of Ferrous Phosphate Hydrate via an Amorphous Intermediate

**DOI:** 10.1021/jacs.3c01494

**Published:** 2023-07-06

**Authors:** Alice Paskin, Thaïs Couasnon, Jeffrey Paulo H. Perez, Sergey S. Lobanov, Roberts Blukis, Stefan Reinsch, Liane G. Benning

**Affiliations:** † GFZ German Research Centre for Geosciences, Telegrafenberg, 14473 Potsdam, Germany; ‡ Department of Earth Sciences, Freie Universität Berlin, Malteserstr. 74-100, 12249 Berlin, Germany; § Federal Institute for Materials Research and Testing (BAM), Richard-Willstätter-Straße 11, 12489 Berlin, Germany

## Abstract

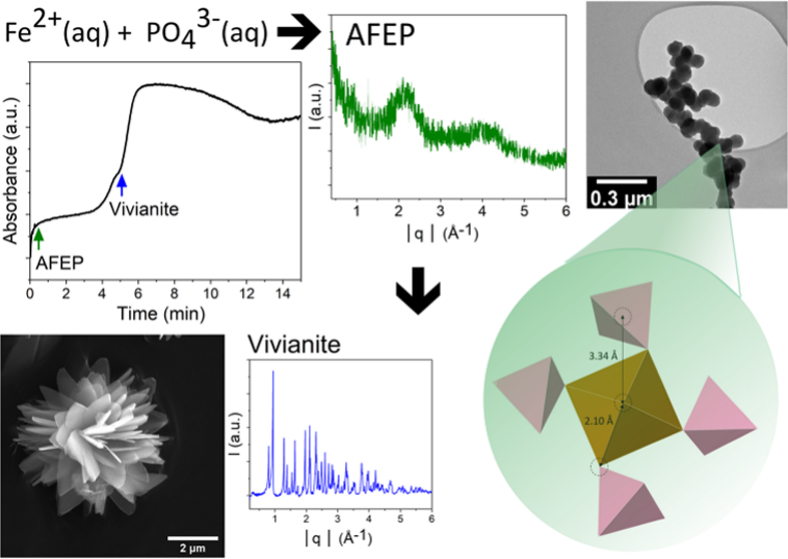

The fundamental processes of nucleation and crystallization are widely observed in systems relevant to material synthesis and biomineralization; yet most often, their mechanism remains unclear. In this study, we unravel the discrete stages of nucleation and crystallization of Fe_3_(PO_4_)_2_·8H_2_O (vivianite). We experimentally monitored the formation and transformation from ions to solid products by employing correlated, time-resolved *in situ* and *ex situ* approaches. We show that vivianite crystallization occurs in distinct stages via a transient amorphous precursor phase. The metastable amorphous ferrous phosphate (AFEP) intermediate could be isolated and stabilized. We resolved the differences in bonding environments, structure, and symmetric changes of the Fe site during the transformation of AFEP to crystalline vivianite through synchrotron X-ray absorption spectroscopy at the Fe K-edge. This intermediate AFEP phase has a lower water content and less distorted local symmetry, compared to the crystalline end product vivianite. Our combined results indicate that a nonclassical, hydration-induced nucleation and transformation driven by the incorporation and rearrangement of water molecules and ions (Fe^2+^ and PO_4_^3–^) within the AFEP is the dominating mechanism of vivianite formation at moderately high to low vivianite supersaturations (saturation index ≤ 10.19). We offer fundamental insights into the aqueous, amorphous-to-crystalline transformations in the Fe^2+^–PO_4_ system and highlight the different attributes of the AFEP, compared to its crystalline counterpart.

## 1 Introduction

The process of crystallization of solid phases in an aqueous solution underlies and controls natural (*e.g*., rock formations,^1^ biomineralization^2,3^), as well as artificial (material synthesis and design^4,5^) systems. Therefore, this fundamental process has received a lot of recognition, particularly in material^6−8^ and environmental sciences.^9−11^ Classical nucleation theory (CNT) states that the thermodynamic barrier of interfacial free energy needs to be overcome by crystalline nuclei to achieve critical sizes, which allows them to grow to macroscopic dimensions via monomer-by-monomer addition.^12^ Although the CNT framework applies to crystallization of iron-bearing phases such as magnetite,^13^ CNT is insufficient in describing many other systems, in which nucleation proceeds via transient amorphous stages, aggregation, and clusters unaccounted for by CNT.^1,14,15^ Ostwald’s law of stages states that the formation of a heterogeneous phase proceeds in multiple stages with each metastable stage having a higher stability than the previous stage, a process that continues until the most thermodynamically stable end state is reached.^16,17^ As a consequence, several studies have recently shown that the formation of thermodynamically stable phases follows a nonclassical pathway. Among these are the calcium and magnesium carbonate (Ca–Mg–CO_3_) phases calcite^18^ and dolomite,^19^ or calcium phosphate (apatite),^20^ which have all been shown to crystallize via the dehydration of amorphous calcium carbonate (ACC)^21−23^ and amorphous calcium phosphate (ACP)^24−26^ precursors. The nucleation of hydroxyapatite (HAP) via ACP and the formation of CaCO_3_ polymorphs (aragonite, vaterite, and calcite) from ACC have been shown to have substantial implications for biomineralization.^2,26,27^*In situ* TEM studies have documented that amorphous-to-crystalline transformations in the calcium carbonate and calcium phosphate systems can occur simultaneously via multiple concurrent pathways.^28−30^

The dihydrous gypsum (CaSO_4_·2H_2_O) end member of the Ca–SO_4_ system was shown to form via the hydration^31^ and particle-mediated rearrangement of anhydrous nanocrystalline precursor.^32^ It is thus clear that nonclassical crystallization of thermodynamically stable phases can proceed via hydration or dehydration and that the pathway is dependent on the interplay between thermodynamic and kinetic factors. Nevertheless, for many sparingly soluble salt systems, a comprehensive elucidation and structural analysis of kinetically controlled crystallization stages and possible intermediate phases remain scarce.

One such understudied phase is Fe_3_(PO_4_)_2_·8H_2_O (vivianite), the most thermodynamically stable phase (pK_sp,25°C_ = 35.416)^33,34^ in the Fe–PO_4_ system. Vivianite is a two-component hydrated solid phase that acts as a crucial mineral controlling the iron and phosphorus element cycles^35,36^ in modern and ancient ferruginous (Fe^2+^ rich) anoxic systems worldwide.^34,37−39^ In addition, vivianite has a major economical relevance in phosphorus recovery from wastewater^40−43^ and the removal of harmful contaminants like lead from the environment.^44^ Vivianite is also a precursor in the synthesis of LiFePO_4_, a material used for battery (cathode) applications.^45^ Yet, the mechanisms controlling vivianite nucleation and crystallization remain unstudied.

In the present study, we unraveled the fundamental nucleation and growth pathway from solution leading to the formation of crystalline vivianite with a focus on the changes in aqueous and solid-state chemical environments. By combining *in situ* ultraviolet–visible (UV–vis) spectrophotometric monitoring of the reaction progress with multiple complementary *ex situ* solution and solid analyses, we characterized the formation and transformation of all reaction stages as well as the changes in structure and bonding of iron and phosphorus and the fate of water in the formed solid phases. This allowed us to derive a mechanism for the crystallization of vivianite.

## 2 Experimental Section

### 2.1 Synthesis and Thermochemical Modeling

All experiments were performed at room temperature inside an anaerobic chamber (Coy Laboratory Products, Inc.) under a gas atmosphere of 97% N_2_ and 3% H_2_. Labware used for synthesis was soaked in 10% HCl solution overnight and rinsed with ultrapure water (∼18.2 M Ω·cm resistivity). All experimental solutions were prepared from ultrapure water that had been degassed by purging with CO_2_-free Ar gas and heating (at 80 °C) for at least 5 h.

Fe_3_(PO_4_)_2_ phases were synthesized in a perfluoroalkoxy alkane reactor by mixing equimolar (0.01 mol L^–1^) solutions of ferrous ammonium sulfate (Fe(NH_4_)_2_(SO_4_)_2_·6H_2_O, 99.95%, Alfa Aesar GmbH) and a mixed sodium potassium phosphate reagent ([HPO_4_^2–^] = 0.01 mol L^–1^) with the latter buffered at pH 7.2 (see Section S1, Supporting Information for phosphate reagent preparation protocol). The experimental pH was continuously recorded every 1 s using a multipurpose data logger board, “DrDAQ” (Pico Technology, Cambridgeshire, UK), equipped with a pH probe, calibrated with NIST pH buffers. The resulting suspension was stirred at 300 r.p.m on a stirrer plate throughout the reaction. After fixed time periods (30 s and 6 min), an aliquot of the solution was removed and filter-quenched immediately by fast vacuum filtration through a 0.2 μm membrane filter (nucleopore polycarbonate membrane). The solids were rinsed with ultrapure water and isopropanol (≥99.5%, Sigma-Aldrich) to remove excess salts and water. The time taken to filter the solution was ≤30 s. The resulting solids were allowed to dry for 5 h inside the anaerobic chamber and transferred to an air-tight crimp-sealed vial for storage until further characterization.

Thermodynamic and geochemical modeling (Section S2, Supporting Information) of the system using the ionic concentrations, pH, and temperature of the experiments were carried out using the PHREEQC program^46^ (Version 3) via the Thermoddem database.^47^

### 2.2 *In Situ* UV–Vis Spectrophotometry

Time-dependent changes in UV–vis absorbance (turbidity) upon mixing of the solutions described above were monitored using an Evolution 220 spectrophotometer (Thermo Fischer Scientific) equipped with an in-built stirrer/temperature control unit (Peltier accessory) and a 3.5 mL septa-sealed quartz cuvette (Sealable Cell 10 mm, Hellma GmbH), which was under a continuous argon gas flow to maintain anaerobic conditions. The cuvette was filled with ultrapure water (∼18.2 M Ω·cm resistivity) and measured as a blank. UV–vis measurements were started with 1.5 mL of the aqueous phosphate buffer reagent (0.01 mol L^–1^) equilibrated inside the cuvette in the UV–vis spectrophotometer at room temperature. The UV–vis absorbance was recorded at a fixed wavelength of 450 nm as that wavelength corresponds to the lowest absorbance intensity for the initial solution. Once a stable reading was attained, 1.5 mL of the ferrous ammonium sulfate solution (0.01 mol L^–1^) was quickly injected into the cuvette under constant stirring and under an argon flow. The absorbance was then recorded at a rate of 1 frame/s with experiments run for 16 min till no further changes were observed.

### 2.3 Characterization

#### 2.3.1 Inductively Coupled Plasma Optical Emission Spectroscopy (ICP-OES)

To determine the dissolved ion concentrations during the experiments, an aliquot of the reaction mixture was filtered through a syringe filter (0.1 μm, PTFE) and collected in acid-cleaned PP tubes. The liquid phase was acidified by conc. HCl (AristaAR, VWR) and stored at 4 °C until analysis.

To determine the resulting solid composition (i.e., Fe/P ratio), powdered solid samples were digested using conc HCl in acid-cleaned flasks. Elemental concentrations in the acidified samples were analyzed using a Varian 720ES ICP-OES. The samples were spiked with 1 mg g^–1^ of cesium as an ionization buffer and scandium (1 μg g^–1^) as an internal standard. Ionic concentrations were evaluated using the emission wavelengths of 261.382 nm (Fe) and 213.618 nm (P), respectively. The determined limits of detection (LoD) for iron and phosphorus are 0.014 and 4 μg L^–1^, respectively (refer to Table T1 in the Supporting Information for quality control (QC) and analytical uncertainties).

#### 2.3.2 Powder X-ray Diffraction (XRD) and Pair Distribution Function (PDF) Analyses

The XRD samples were prepared inside the anaerobic chamber by grinding the dried powders with a mortar and pestle and transferring them into a glass capillary (Hilgenberg 4007805, 0.5 mm) sealed with a wax plug (Cristaseal Sealant Tray, Hawksley & Sons Ltd.) to prevent oxidation. XRD patterns were measured in a Debye–Scherrer geometry on a STOE STADI P (STOE & Cie GmbH, Germany) diffractometer operating at 40 kV and 40 mA, using Ag K_α_ radiation (λ = 0.55941 Å), equipped with a curved Ge(111) monochromator and two DECTRIS MYTHEN2 R detectors with a 0.015° step size and 1500 s per step. The 2θ values ranged from 1 to 70°. An empty capillary was measured as the background, under the same experimental conditions. The background patterns were subtracted from the experimental patterns prior to plotting and analysis. XRD data handling was performed using the STOE WinXPOW software (Version 3.21.2) and OriginPro (2021) software (OriginLab Corporation, Northampton, MA). The calculated XRD patterns were obtained from the respective crystallographic information file (cif)^48^ and using the Vesta software.^49^

*Pair distribution function* (PDF) analysis was done with the PDFGETX2 software^50^ and using the powder XRD patterns measured up to 140° (2θ). Data treatment included background subtraction (0.5 mm empty capillary), polarization (Ge(111), *d* = 3.226 Å), Compton, multiple, diffuse scattering, and absorption (μt = 0.3) corrections. PDFs were generated up to a maximum *r* of 50 Å from Fourier transform of the *Q*[*S*(*Q*) – 1].

#### 2.3.3 Scanning Electron Microscopy (SEM)

Samples were prepared inside the anaerobic chamber by suspending ∼5 mg of a dried solid sample in degassed isopropanol and drop-casting onto SEM stubs. The isopropanol was allowed to evaporate, and then the samples were removed from the anaerobic chamber and immediately carbon-coated (∼20 nm layer) using a high-vacuum sputter coater (BAL-TEC MED 020 Leica Microsystems) prior to SEM analyses on an FEI Quanta 3D field-emission-SEM instrument at 20 kV. Particles were imaged, and elemental maps were recorded using energy-dispersive X-ray spectroscopic analyses (SEM-EDS).

#### 2.3.4 Transmission Electron Microscopy (TEM)

Inside the anaerobic chamber, aliquots of the reaction mixture were quenched at 30 s, 100 s, 6 min (SI 10.19), 20 min, and 48 h (SI 7.16). These samples were drop-casted onto lacy carbon-coated copper grids that were deposited on the filtration frit of a vacuum filtration assembly. The samples on the grids were rinsed with ultrapure water and isopropanol to remove salts and subsequently dried under vacuum. They were then transferred from the anaerobic chamber to a TEM sample holder and inserted, within 1 min, into the TEM airlock and set under vacuum. Micrographs and analyses of the samples were acquired using a TECNAI F20 XTWIN TEM operated at 200 kV with a field-emission gun electron source and a Gatan Imaging Filter (GIF) Tridiem EDS X-ray analyzer. Imaging, selected area electron diffraction (SAED), and scanning-TEM coupled to EDS (STEM–EDS) were used to analyze particle morphology, crystallinity, and qualitative chemical composition of the samples. The TEM image analysis was performed using the Image J (U.S. National Institute of Health) and TIA (FEI Company and digital micrograph, Gatan Inc.) software packages.

*Fourier transform infrared spectroscopy (FTIR)* spectra of the dried solids were acquired using the attenuated total reflection (ATR) mode on a Nicolet iS5 spectrometer (Thermo Fischer Scientific) with an iD7 diamond ATR accessory and KBr beam optics. FTIR spectra were acquired within a range of 400–4000 cm^–1^, and 16 scans were averaged with a resolution of 4 cm^–1^. Spectra were analyzed using the Thermo-Nicolet OMNIC version 1.02 software package (Thermo Fisher Scientific Inc.).

#### 2.3.5 Thermal Analyses

Thermogravimetric measurements (TGA) were used to determine the water content of the samples. Data were acquired on a thermobalance SETARAM TAG 24 (Setaram, Caluire, France) system. The powdered samples were stored under nitrogen until insertion into open platinum crucibles (100 μL). Measurements were conducted under an argon flow (35 × 10^–5^ L s^–1^) after repeated evacuation cycles (∼3 × 10^–1^ mbar) at a heating rate of 10 K/min up to a maximum temperature of 500 °C. The data were analyzed using the OriginPro (2021) software (OriginLab Corporation, Northampton, MA).

#### 2.3.6 X-ray Absorption Spectroscopy (XAS)

The XAS data was collected at the Fe K-edge (7112 eV) on the P65 undulator beamline of the Deutsches Elektronen-Synchrotron (HASYLAB, DESY PETRA III, Hamburg, Germany) operated at 6.0 GeV with a 100 mA current in a multibunch mode. Powdered samples were mixed with cellulose (2.5% Fe) and pressed into ∼1 mm thick and 13 mm Ø pellets, which were sealed inside the anaerobic chamber with a 25 μm thick Kapton tape that has a low O_2_ diffusivity.^51^ Samples were transferred and transported to DESY inside crimped vials.

At DESY, these vials were reinserted and stored in a glovebox until just prior to measurement when they were transferred into the liquid helium flow cryostat (Oxford Instruments), which allowed for the samples and reference compounds (synthetic γ-FeO(OH)^52^ and Fe(OH)_2_^53^) to be maintained at 20 K and 10^–6^ mbar and thus prevent oxidation and photoreduction under the beam during the XAS measurements.

Incoming photon flux energy was modulated with a Si(111) double crystal monochromator, with an energy resolution of ∼0.7 eV at the Fe K-edge and a beam size of 0.3 × 1.5 mm^2^. The effective suppression of higher harmonics was achieved using Si-plane mirrors. The data were collected from −150 eV below the edge to +1000 eV above with a scan energy increment of ∼0.6 eV in a continuous mode. The time for each spectrum was 240 s, and an average of 5 scans was used for the analysis. Spectra of reference compounds and samples were acquired in transmission mode, concomitantly with the spectrum of an Fe foil for energy calibration and alignment. The first inflection point in the first derivative of the adsorption threshold of the Fe foil was calibrated at 7112 eV. Incident and transmitted X-ray intensities were recorded using ion chambers with a path length of 5 cm. The ion chambers were filled with a gas mixture of N_2_, Ar, and Kr to approximately obtain 15, 50, and 100% absorptions for the incident beam I_0_, the transmitted beam I_T_, and the beam transmitted through the reference foil I_T2_, respectively. X-ray absorption near-edge structure (XANES) data handling and edge analyses were done on Athena software.^54^ Prepeaks were obtained by programming and subtracting a fitted arctangent function on OriginPro (2021) software (OriginLab Corporation, Northampton, MA). The prepeak components were deconvoluted and fitted using Fityk software.^55^ Extended X-ray absorption fine structure (EXAFS) modeling was done by fitting Fe–O/P paths on SIXpack software using IFFEFIT database.^56^

#### 2.3.7 Optical Spectroscopy (visible to near-infrared)

Optical spectroscopy was performed to characterize the *d–d* electronic transition bands in the solid phases. Data were recorded from 7000 to 18 000 cm^–1^. The spectra were collected on a Vertex 80v spectrometer (Bruker) with exchangeable UV–vis and near-IR beam-splitters. Samples were powdered by a mortar and pestle and suspended in epoxy resin, inside the anaerobic chamber, and smeared on a glass slide for measurements. Then, the sample was taken out of the anaerobic chamber and transported to the instrument for data collection. Optical reference was measured through the glass slide and epoxy resin. UV bands were deconvoluted and fitted using Fityk software for pseudo-Voigt profiles.^55^

### 2.4 Stability of Initial Precipitates

The initial precipitates were filter-quenched after 30 s of mixing equimolar (0.01 mol L^–1^) solutions of ferrous ammonium sulfate (Fe(NH_4_)_2_(SO_4_)_2_·6H_2_O, 99.95%, Alfa Aesar GmbH) and a mixed sodium phosphate reagent ([HPO_4_] = 0.01 mol L^–1^) at pH 7.2 (see Section S1, Supporting Information), as described in Section 2.1. The solids were characterized by IR spectroscopy, and ∼30 mg of aliquot of each solid was transferred into a crimped vial with 50 mL of ultrapure water (∼18.2 M Ω·cm resistivity). The mixture was crimp-sealed and kept at room temperature inside the anaerobic chamber for 5 d. An aliquot of the suspension was filtered and dried (see Section 2.1 for filtration setup) inside the anaerobic chamber. IR and SEM analyses of the resulting products were performed after 24 h and 5 d, respectively.

## 3 Results and Discussion

### 3.1 Detection of an Amorphous Iron Phosphate Intermediate during the Crystallization of Vivianite

Using the available thermodynamic data for vivianite (solubility product *K*_sp_ at 25 °C: 1.75 × 10^–35^),^33^ at the equimolar ionic concentrations ([Fe^2+^] = [HPO_4_^2–^]: 50, 0.005 and 5 × 10^–4^ mol L^–1^ in the mixed solutions), our computational modeling showed that under the given conditions our experimental solutions were supersaturated exclusively with respect to vivianite (saturation index (SI) = 10.19; for SI calculations, see Section S2, Supporting Information).

**Figure 1:**
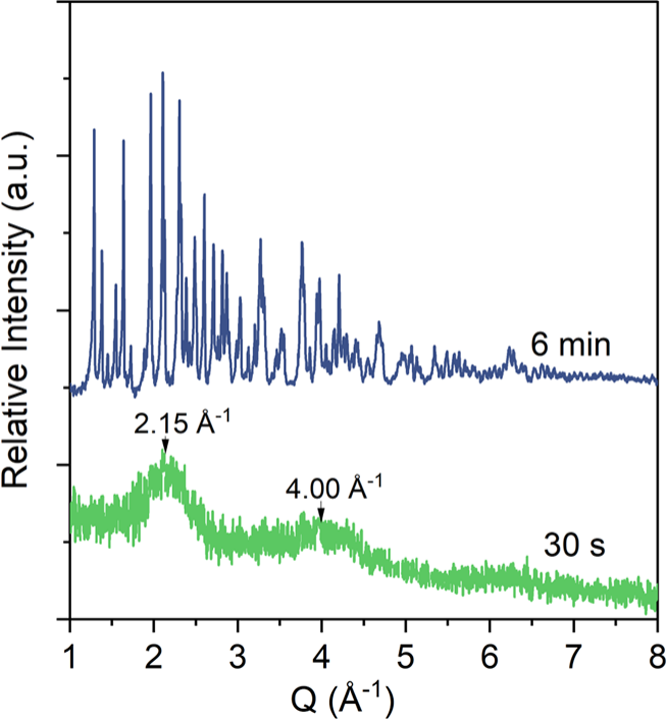
Powder X-ray diffraction patterns of filter-quenched solids collected from the reaction mixture after 30 s (green) and 6 min (blue); +30 s for filtration; SI vivianite is 10.19.

However, immediately upon mixing of the equimolar solutions at SI 10.19 ([Fe^2+^] = [HPO_4_^2–^]: 0.005 mol L^–1^), we observed the formation of a pale green suspension that transformed with time into bluish-white precipitates, which, toward the end of the experiments (15 min), settled at the bottom of the PFA reactor. When the pale green suspension was filter-quenched after 30 s, the powder XRD analyses revealed a poorly ordered phase, evidenced by two broad peaks centered at *Q* values of 2.15 and 4.00 Å^–1^ (Figure 1).

Filter-quenching the mixed solutions after 6 min, the XRD pattern evidenced sharp Bragg reflections assignable to pure crystalline vivianite (Figure 1). Using the literature *cif* file for vivianite,^48^ we derived a calculated vivianite pattern and compared it with the spectra from our synthetic vivianite produced after 6 min and found a good match for all peaks (Figure F1, Supporting Information).

**Figure 2:**
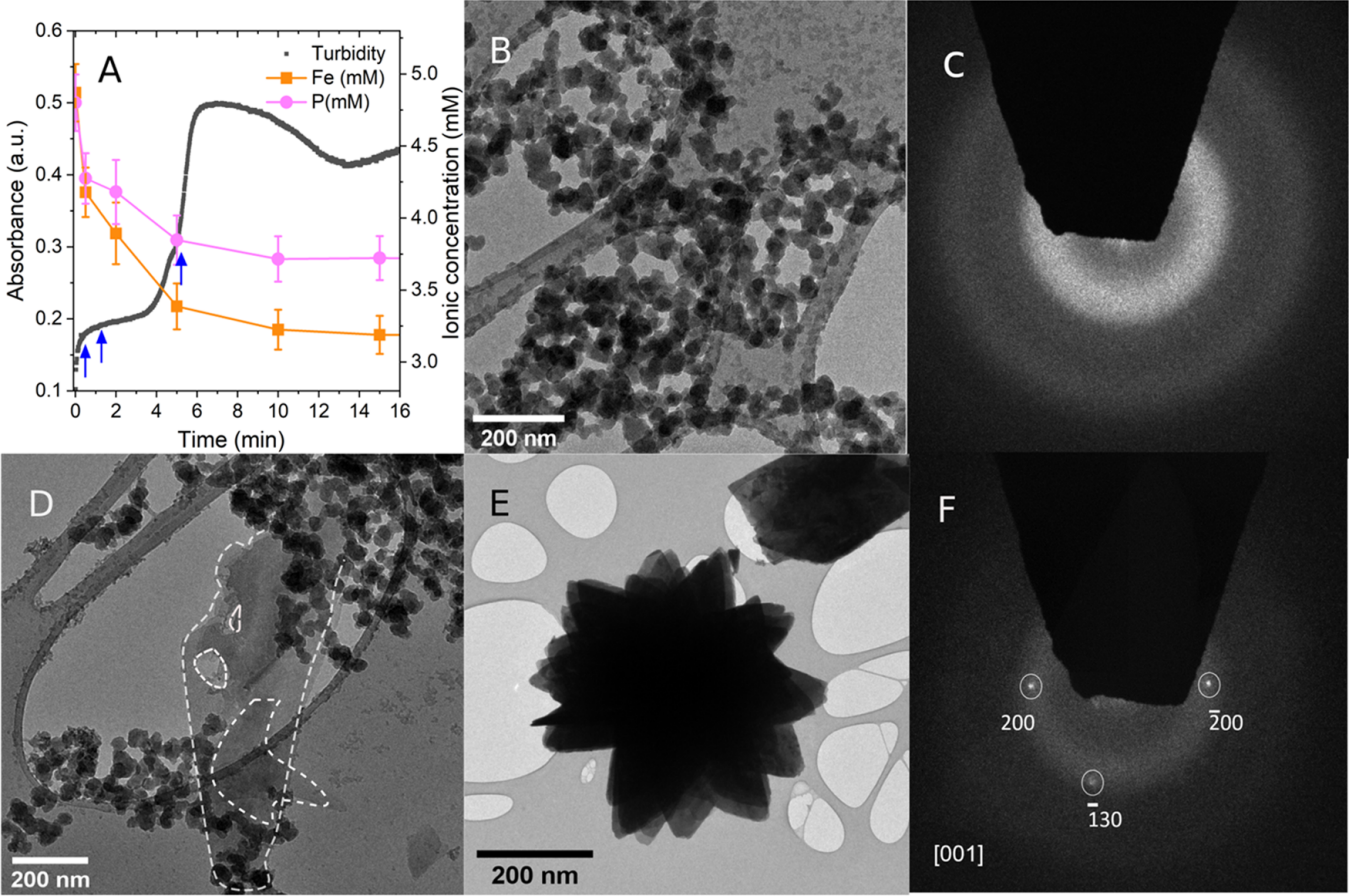
(A) *In situ*, time-resolved turbidity (black curve) and *ex situ* ICP-OES analyses of dissolved species (Fe, orange; P, pink points) during a vivianite precipitation experiment at SI 10.19, with error bars representing values from three repeat measurements. Arrows in blue represent sampling points for TEM analysis; (B) TEM micrograph of solids after 30 s of reaction, showing aggregated nanoparticles; (C) associated SAED pattern of panel B showing diffused scattering features indicating an amorphous phase; (D) TEM micrograph after 100 s of reaction showing both the amorphous nanoparticulate aggregates and the initial thin vivianite platelets (dashed white outline); (E) TEM micrograph of solids after 6 min showing radial platy florets of vivianite; and (F) associated SAED pattern of panel E indexed along the [001] zone axis.

The increase in turbidity (measured as absorbance) in our *in situ* UV–vis spectrophotometric experiments evidenced a stepwise increase that can be equated with the multistage formation of solid phases (Figure 2A, black trace). Contrary to the sigmoidal shape of UV–vis spectrophotometric curves reported for single-step mineral crystallization reactions,^9,57^ the turbidity curve clearly suggested intermediate stages of nucleation and crystallization (Figure 2A).

Immediately upon mixing, the turbidity increased, indicating the instantaneous formation of a solid phase with higher scattering than the initial solutions. After ∼30 s, a first plateau was reached (the 1st blue arrow in Figure 2A) and the turbidity remained constant for up to ∼4 min. A second drastic increase in turbidity clearly suggested a secondary stage in the phase formation reaction followed by a maximum having been reached at ∼7 min with a shoulder on the sharp increase. UV–vis spectrophotometric curves having an initial plateau and a second marked increase in turbidity have been reported for the crystallization of the ferrous carbonate mineral, siderite (FeCO_3_).^10^ In their study, Mulders et al. showed that the initial increase of absorbance was a consequence of nucleation of a nanoscale amorphous ferrous carbonate precursor, followed by the second marked increase in turbidity due to the transformation of the amorphous precursor into crystalline siderite.^10^

In our work, the plateau and sudden increase observed during the precipitation also suggested the presence of an intermediate phase immediately after mixing and a secondary transformation to another phase between 4 and ∼7 min when a 2nd plateau is reached. The patterns were reproducible in repeat experiments. In all cases, after the 2nd plateau was reached, a gradual decrease in turbidity was observed, a decrease explainable by the gravitational settling of the growing precipitates. Such decreases, linked to settling, are common in turbidity patterns and have, for example, been reported for calcite crystallization via amorphous calcium carbonate (ACC), where the larger stable calcite crystals settled following their transformation from ACC.^6^ We cross-correlated our turbidity observations with *ex situ* analyses of the changes in dissolved iron and phosphorus concentrations during the precipitation at different time points (Figure 2A, pink, orange points). The fast-initial turbidity increase was mirrored by a fast-initial decrease in dissolved iron and phosphate concentrations upon mixing. This confirmed the instantaneous formation of an iron phosphate phase. However, the first initial drop in ion concentrations did not precipitate all ions and did not reach^33^ vivianite solubility product values ([Fe^2+^] = 1.31 × 10^–7^ mol L^–1^, [PO_4_^3–^] = 8.76 × 10^–8^ mol L^–1^). After ∼5 min, a second more minor decrease in Fe or P concentrations was observed, and apparent equilibrium was nearly achieved at the same time as the maximum in turbidity was reached. The two different rates of changes in aqueous Fe and P concentrations indicate the initial instantaneous nucleation of an Fe–P phase, which in the 2nd stage sequestered additional Fe and P ions from the surrounding medium to form the solids with different scattering, as evidenced by the 2nd increase in turbidity after ∼4 min (Figure 2A).

Analysis of the acid-digested solids retrieved from the experiments after 30 s revealed an Fe/P ratio of 1.41 ± 0.01, which matches the stoichiometric composition of a solid with formula Fe_3_(PO_4_)_2.12_. Based on this stoichiometry, one could infer the presence of an Fe^2+^ phosphate. However, since the ICP-OES technique analyzes the total Fe content in the digested precipitates and cannot detect the partial oxidation of Fe, we verified the oxidation state of Fe by synchrotron Fe K-edge X-ray absorption spectroscopy (XAS). Not surprisingly, the analysis of the crystalline end product vivianite yielded an Fe/P ratio of 1.45 ± 0.01, matching vivianite’s expected chemical composition.

The rapidly nucleated poorly ordered phase (green XRD pattern in Figure 1) remained stable for a few minutes prior to the secondary increase in turbidity. If its transformation would occur by dissolution and subsequent reprecipitation to vivianite, the turbidity curve should have indicated a temporary decrease in absorbance before the start of the 2nd rapid absorbance increase at ∼4 min. This was not observed, although from our XRD data (blue pattern in Figure 1), it is clear that crystalline vivianite was formed. Although we cannot exclude some dissolution, our data seem to indicate that the amorphous precursor did not (or only partially) dissolve prior to vivianite crystallization. For monodisperse suspensions, particle sizes can theoretically be correlated to turbidity measurements through Mie scattering approximations.

However, in our experiments, this is difficult due to the fast changes in the physical and chemical properties of our rapidly evolving suspension (i.e., changes in solution and solid compositions, refractive index, particle density, and shape)—all parameters that are difficult to control, especially for fast reactions, such as this. However, we could derive particle morphologies and sizes as a function of time from TEM micrographs of *ex situ* filter-quenched solids. At the time points indicated by arrows in Figure 2A (30 s, 100 s, and 6 min), samples were collected and analyzed. The corresponding TEM micrographs (Figure 2B–F) revealed that the precipitates obtained after 30 s were nearly spherical nanoparticles of a fairly monodisperse particle size distribution of ∼50 nm diameters, which were most often present as aggregates (Figure 2B). Associated SAED patterns showed only diffuse rings characteristic of an amorphous material (Figure 2C). The STEM–EDS analysis of these particles only evidenced the presence of iron, phosphorus, and oxygen in the patterns, without any other impurities (Figure F2, Supporting Information).

The TEM micrographs of solids acquired within the time frame of the 1st plateau of the turbidity curve (at 100 s) revealed not only aggregated nanoparticles but also very thin platelets (white dotted outline in Figure 2D). To unravel the variable structural order within this sample, we used high-resolution TEM (HR-TEM) coupled to fast Fourier transform (FFT). The data indicated areas of variable structural order within the sample (Figure F3, Supporting Information). The very thin platelets (Figure 2D) were crystalline vivianite (Figure F3, Supporting Information), yet all nanoparticles surrounding the poorly formed vivianite platelets were amorphous (Figure F3, Supporting Information). This suggests that the first, thin vivianite platelets may already form immediately after the instantaneous nucleation and stabilization of the amorphous nanoparticles as evidenced by the 1st plateau in the turbidity pattern (Figure 2A).

On the other hand, the precipitates after 6 min consisted of larger, radial plate-shaped crystalline florets ranging between 0.5 and 5 μm in diameter (Figure 2E). The associated SAED pattern was indexed and matched crystalline vivianite (along the [001] zone axis; Figure 2F).

Our *in situ* turbidity data evidenced a nearly constant trend of ∼2 min (1–3 min), despite the TEM data indicating the concomitant presence of minor vivianite and the initiation of the amorphous-to-crystalline transformation during the 1st plateau (100 s). This particular trend can be understood by a kinetic steady-state approximation stating that a constant (time-independent) concentration of a reactive species can be approximated from varying concentrations of the reagents, assuming equal rates of its formation and consumption.^58^ Therefore, the formation rate of this intermediate and its rate of transformation to the first thin vivianite platelets can be assumed as approximately equal during the transition period, as evidenced by a nearly constant turbidity trend between 1 and 3 min. When all the solid phases have precipitated, the steady state is disrupted and the reaction is driven rapidly to the more thermodynamically stable end state. This is cross-correlated by all the presented observations (XRD, *in situ* UV–vis spectrophotometry, ICP-OES, and TEM/SAED), which all confirm that an intermediate nanoparticulate amorphous Fe–P-containing phase formed prior to vivianite crystallization. Since supersaturation is a very important aspect of nucleation and growth, we investigated the kinetic and mechanistic impact of varying vivianite supersaturation in this system.

### 3.2 Effect of Supersaturation

The TEM micrographs (Figure F4, Supporting Information) from an experiment at a lower supersaturation (SI = 7.16) revealed the presence of homogeneous spherical and amorphous nanoparticles of ∼50 nm diameters after 20 min.

**Figure 3:**
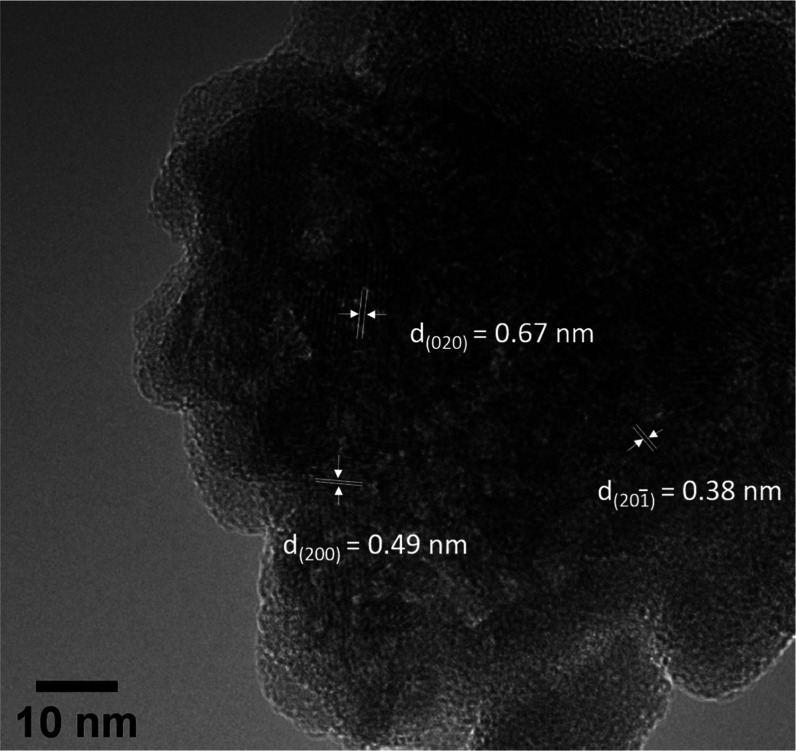
HR-TEM micrograph of aggregated iron phosphate nanoparticles and lattice fringes (*d*_hkl_) corresponding to crystalline vivianite amidst the aggregates. Solids collected at SI 7.16 at 20 min.

The EDX analyses (Figure F5, Supporting Information) of these samples showed the presence of only iron, phosphorus, and oxygen. Compared to SI 10.19 where the majority of the nanoparticles converted to vivianite in ∼5 min, the prolonged presence of the amorphous nanoparticles at this supersaturation (20 min) shows that the lifetime of the precursor increases at a lower SI. HR-TEM micrographs (Figure 3) of the precursor aggregates revealed the presence of dispersed domains of short-range lattice fringes (*d*_hkl_) within these aggregates. These parallel fringes were assignable to the initially forming (020), (200), and (201̅) lattice planes of vivianite. The size range of these domains was ∼10 nm, and their shapes were irregular as shown in Figure 3. These micrographs suggest a transformation process via internal rearrangement, as also inferred for some ACP transformation processes.^24,59,60^ However, since *ex situ* TEM only offers a 2D snapshot of a dynamic process and is not a 3D rendering of a reaction pathway, and sample preparation and imaging itself could also induce artifacts, an absolute conclusion cannot be drawn. The XRD analysis of solids at SI 7.16 at 48 h (Figure F6, Supplementary Information) evidenced sharp Bragg peaks assignable to crystalline vivianite. The corresponding TEM micrographs (Figure F6, Supplementary Information) from the sample at 48 h revealed micrometer-sized vivianite crystals and spherical nanoparticles with surprisingly larger sizes (80–100 nm diameter; Figure F6, Supplementary Information, bottom image) on their surface. If the amorphous nanoparticles dissolved prior to vivianite crystallization, their sizes should have decreased with time, as observed for ACP, in some cases.^61^ However, their sizes increased with time (Figure F6, Supporting Information) through a possible Ostwald ripening process,^62^ showing that lower supersaturations delay vivianite formation and may indeed favor a solid-state transformation.

An experiment at SI 12.86 was performed to analyze the effect of a higher supersaturation. The XRD analyses (Figure F8, Supporting Information) from this experiment showed the precipitation of crystalline vivianite immediately upon mixing and filter-quenching the suspension (∼20 s). This may be caused by rapid homogeneous nucleation and a decrease in the nucleation induction period at very high supersaturations. SEM showed that most of the vivianite crystallized as radial platy florets with faceted crystals (Figure F13, Supporting Information). Noteworthy is nevertheless, where the surface texture of these florets was smooth, within instrumental detection (Figure F13, Supporting Information), indicating that crystal growth may occur via ionic attachment.^63^ Our data show that the overall rate of vivianite formation was greatly enhanced at higher supersaturations (SI ≥ 12.86) and the growth of vivianite occurs via ionic attachment. The kinetics of nucleation, as a function of supersaturation, were also studied by *in situ* pH metric measurement at different vivianite supersaturations at an initial pH of 7.2 (Figure F7, Supporting Information). These results (Section S3, Supporting Information) showed that the rate of nucleation and transformation were kinetically enhanced at higher supersaturations.

In the Ca–PO_4_ system, a solution-mediated dissolution–reprecipitation mechanism of ACP → HAP transformation was initially proposed by Boskey and Posner,^20^ with further corroborating evidence from other sources.^61,64,65^ However, this hypothesis has been challenged by several studies, which proposed a solid-state transformation pathway based on a combination of TEM,^24,59,60^ solid NMR,^66^ and *in situ* XRD, IR^67^ analyses. In some studies, multiple, simultaneous pathways have also been proposed.^30,68,69^ Thus, for the ACP → HAP transformation, it is not surprising that an unequivocal transformation mechanism remains debated. This is similar also in the Ca–CO_3_ system where depending on the reaction followed, dissolution–reprecipitation^23,28,70^ or solid-state transformations^4,71,72^ of ACC → crystalline CaCO_3_ can dominate the transformation process. The data for the amorphous-to-crystalline transformation in the Fe^2+^–PO_4_ system, as well as other sparingly soluble salt systems,^1^ reveal that unique parameters and dominant mechanisms for many such transitions remain unclear. Based on the data presented above (TEM, SEM, XRD), concurrent pathways of vivianite nucleation and growth are possible in this system, and the dominant pathway changes, depending on the supersaturation. The focus of this work is the, previously unreported, amorphous iron phosphate intermediate, which converted to vivianite with time via a predominantly solid-state transformation at SI ≤ 10.19. Since the recovery of the precursor proved to be a challenge, all analyses were based on solids harvested at SI 10.19 at 30 ± 10 s. To understand the factors driving the transformation of the amorphous precursor to vivianite, we probed the nature, chemical composition, and structure of the poorly ordered precursor and crystalline end member vivianite.

### 3.3 Structural Variations in Symmetry and Bonding Environments at the Fe Site

**Figure 4:**
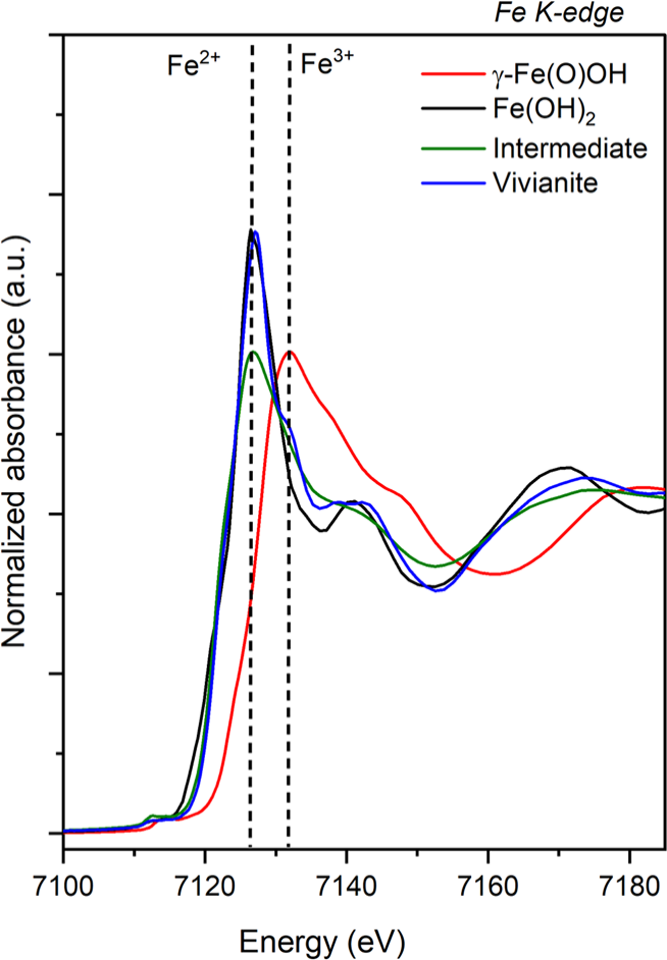
Normalized Fe K-edge XANES spectra collected for synthetic γ-Fe(O)OH (reference for Fe^3+^), Fe(OH)_2_ (reference for Fe^2+^), amorphous intermediate, and vivianite. Dashed lines represent energy edge positions for Fe^2+^ and Fe^3+^ oxidation states.

To accurately determine the Fe oxidation state, possible variations in Fe bonding environments and symmetry in both phases, synchrotron Fe K-edge XAS measurements (XANES and EXAFS), were performed on the solid samples. The Fe K-edge XANES spectra of synthetic vivianite (blue pattern, Figure 4) showed an energy edge position (*E*_o_) of 7121.61 eV based on the position of the maxima in the first derivative plot (Figure F9, Supporting Information), correlating well to the reported literature value of 7121.50 eV for naturally occurring vivianite.^73^ The XANES spectrum of the amorphous intermediate indicated an *E*_o_ value of 7121.29 eV, suggesting the presence of Fe^2+^ as the dominant oxidation state. Comparisons between the *E*_o_ values of vivianite, the poorly ordered intermediate (green and blue spectra, Figure 4), to reference spectra of synthetic Fe^2+/3+^ compounds (black and red spectra, Figure 4), confirmed Fe^2+^ as the dominant oxidation state in both samples. By combining all the so far presented data (XRD, TEM, EDS, ICP-OES, and XANES), we can state that the amorphous intermediate phase is an Fe^2+^ phosphate phase, herewith designated as AFEP (amorphous ferrous phosphate).

**Figure 5:**
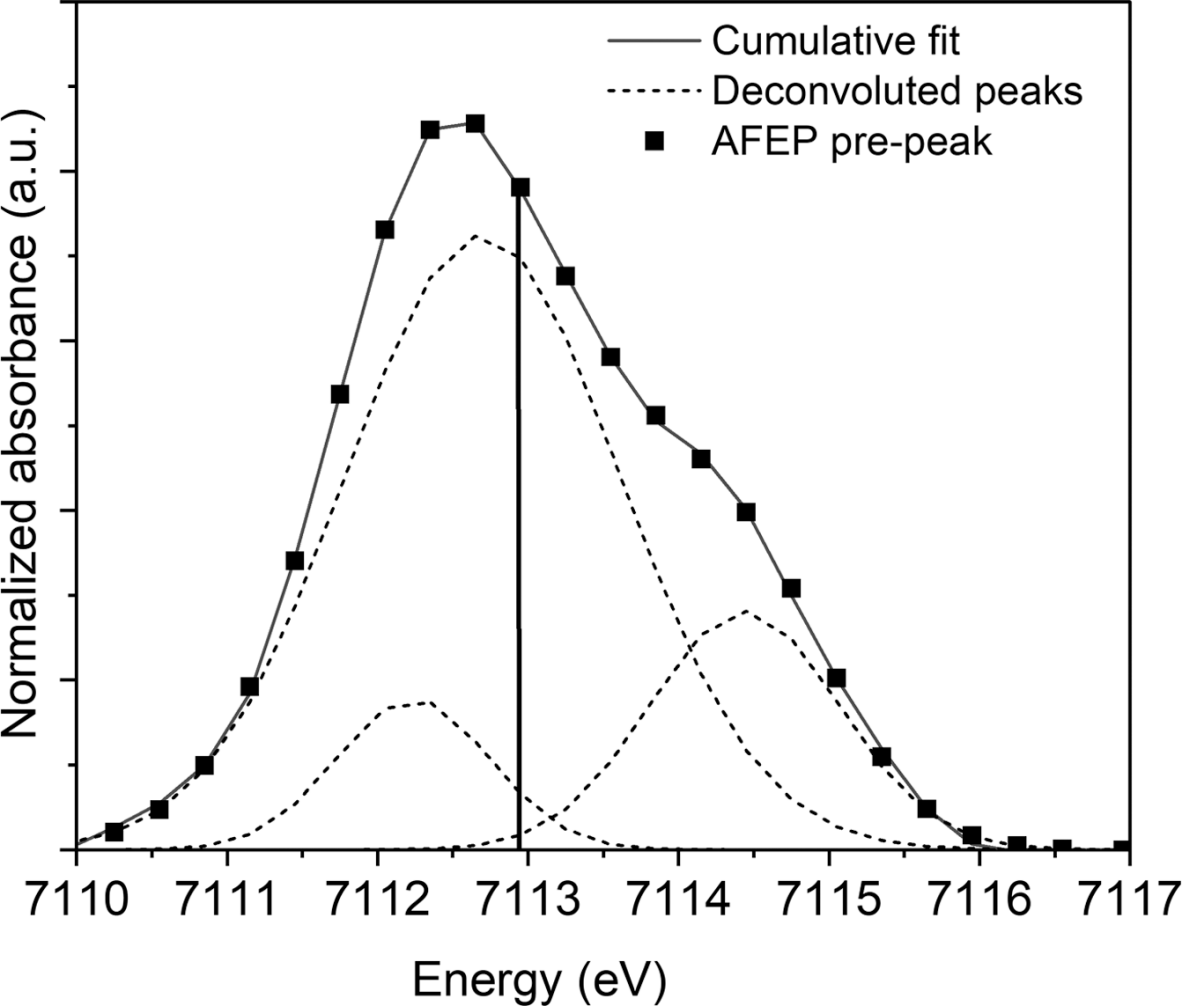
Deconvoluted pre-edge contributions for AFEP (amorphous ferrous phosphate) based on pseudo-Voigt (50:50) function fitting. The vertical solid line represents the centroid position.

Furthermore, the pre-edge feature accounts for 1 s → 3d (quadrupole) or 1 s → 4p (dipole) electronic transitions in the Fe K-edge XANES spectra. The shape and position of the pre-edge depend strongly on the Fe oxidation state, spin, symmetry, and coordination geometry.^74,75^ In the AFEP spectrum, the pre-edge feature was extracted by interpolating the background and modeling the contribution of the edge jump to the pre-edge using a spline function several eV before and after the pre-edge (Figure F10, Supporting Information). This method for pre-edge analysis has been used by Wilke et al. for studying oxidation states and coordination of iron-bearing mineral phases.^74^ We deconvoluted the pre-edges into pseudo-Voigt components with equal Gaussian and Lorentzian (50:50) contributions and fitted the spectra for octahedrally coordinated Fe^2+^ compounds with three components based on theoretical predictions, symmetry considerations, and 3d–4p orbital hybridizations.^76,77^ The position of the pre-edge “centroid” or weighted average of the fitted peaks is centered at approximately 7113.00 eV for Fe^2+^ and at 7113.50 eV for Fe^3+^ mineral phases.^74,75^ A centrosymmetric and nondistorted Fe octahedron gives rise to three predicted features directly in the raw pre-edge data. This peak deconvolution revealed features at 7112.35, 7112.65, and 7114.45 eV (Figure 5). The weighted average or centroid of the deconvoluted peaks was extracted via integrated peak area percentages and the maxima of each component. The centroid was calculated to be at 7112.95 eV, which is positioned well within the range for the Fe^2+^ oxidation state,^75^ reinforcing the edge-energy (E_o_) XANES analysis. The pre-edge fitting of AFEP indicated a nearly nondistorted octahedral high-spin (S = 2) Fe^2+^ center based on the fitting results.

**Figure 6:**
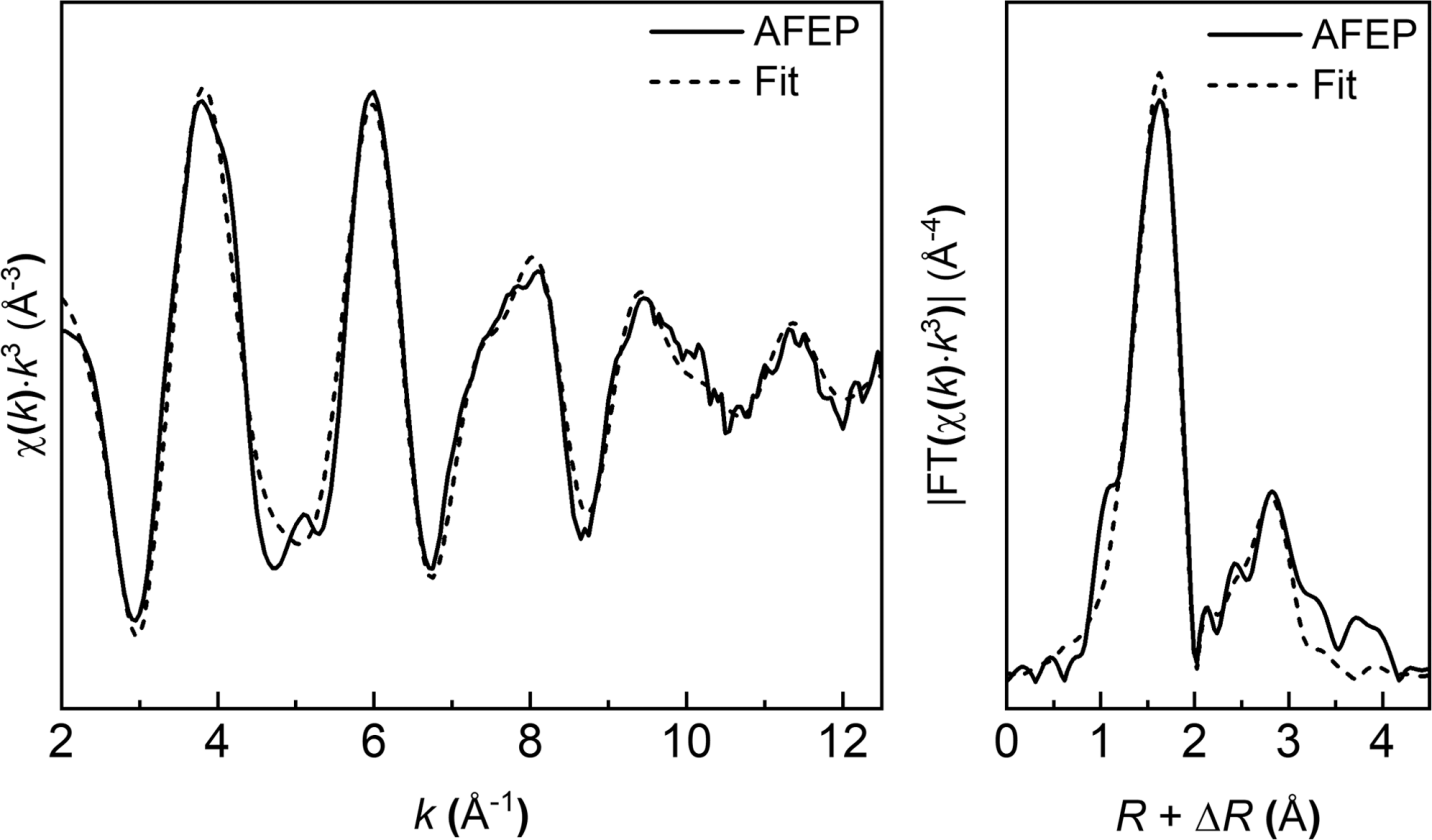
Fe K-edge-normalized, background-subtracted, *k*^3^-weighted EXAFS spectra and Fourier transforms (FTs) of the AFEP sample. Solid lines, experimental spectra; dashed lines, fitted spectra.

**Figure 7:**
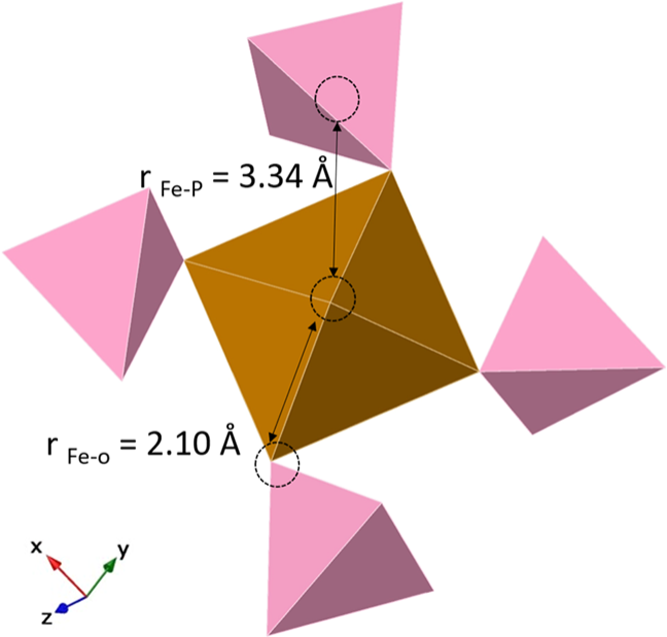
Average local structure of AFEP with four PO_4_ tetrahedra bonded to an FeO_6_ octahedron; bond distances modeled via EXAFS first and second shell-fits. Bond distances are shown in Angstrom (Å) units.

Predictable, marked differences were observed in the EXAFS spectra of vivianite and AFEP, with differences noticeable particularly in their FT-EXAFS spectra, indicating a variable Fe coordination and composition (Figure F9, Supporting Information). To unravel the local structure of AFEP and study its coordination environment, shell-by-shell fittings for Fe–O and Fe–P paths were performed on the Fe K-edge data (Figure 6 and Table T3, Supporting Information). Background-subtracted, normalized, and *k*^3^-weighted EXAFS spectra and Fourier transforms (FTs) of AFEP and the fits (Figure 6) revealed the first neighbor contribution in AFEP being the Fe–O atomic correlation with an interatomic distance (*R*_Fe-O_) of 2.10 ± 0.01 Å and a coordination number (CN) of ∼6. The second neighbor contribution was assigned to the Fe–P atomic correlation with an *R*_Fe-P_ of 3.34 ± 0.01 Å and a CN_Fe-P_ of ∼4. This suggests an average local structure wherein four PO_4_ tetrahedra and two H_2_O molecules are octahedrally coordinated to the central Fe^2+^ atom. We used these data to model the local structure of AFEP based on the EXAFS fitting results (Figure 7). We can compare this with data from Mikutta et al. who modeled and fitted the first shell of EXAFS spectra of amorphous ferric phosphate and obtained an average Fe–O distance of 1.97 Å.^78^ Unsurprisingly, the higher oxidation state of Fe in the ferric phosphate leads to a shortening of the Fe–O bond and thus the shorter average bond distance as compared to 2.10 Å in our amorphous ferrous phosphate (AFEP).

**Figure 8:**
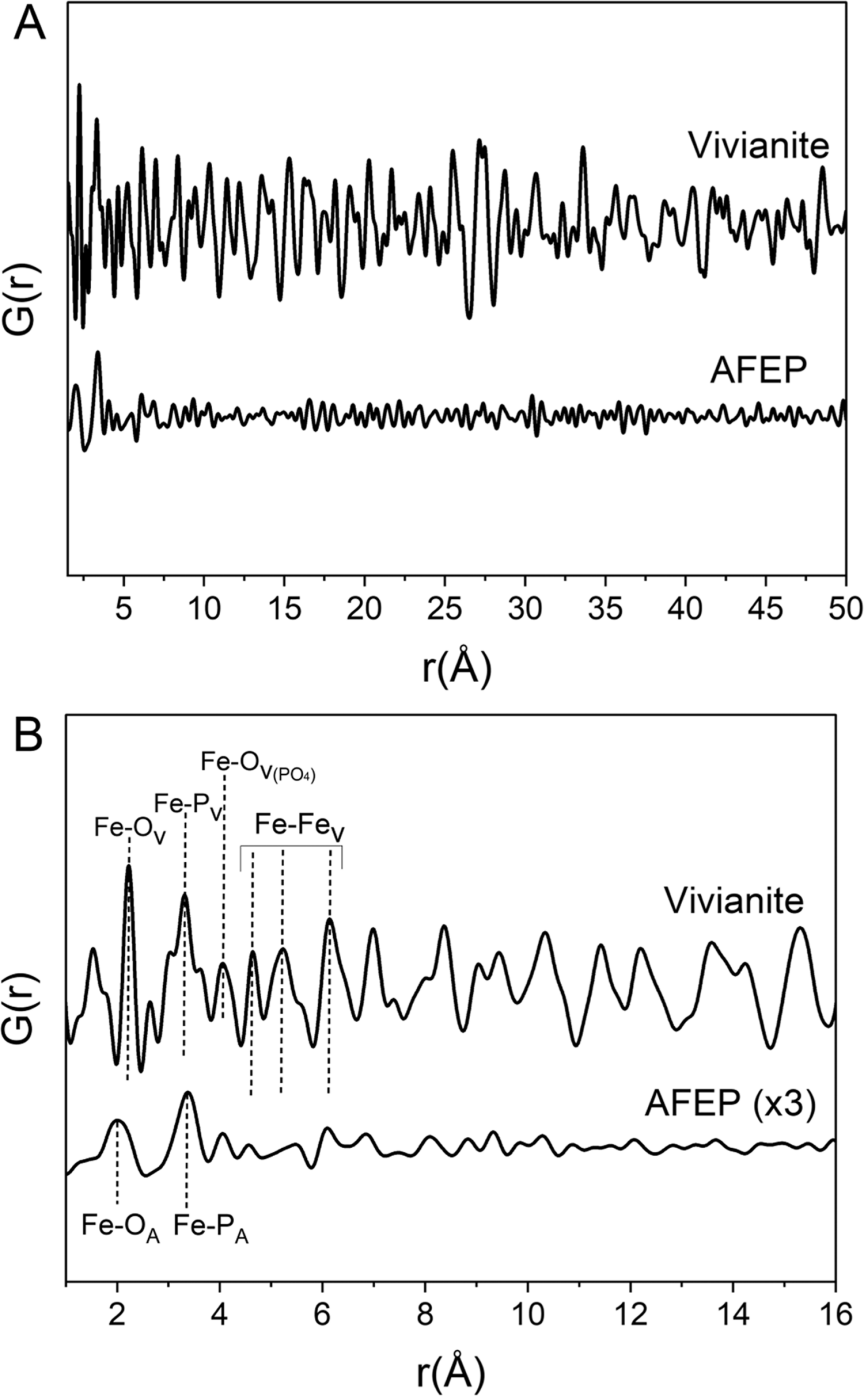
(A) *G*(*r*) for vivianite and AFEP. The *G*(*r*) decays rapidly with increasing *r*-values for AFEP, reflecting smaller coherently scattering domains than vivianite. (B) Zoom of lower *r*-values. Peak intensities for AFEP have been tripled (×3) so that small peaks are discernible. Subscripts: A for AFEP and V for vivianite.

We cross-confirmed the local ordering of both phases through the reduced PDF (*G*(*r*)) analysis of AFEP and vivianite and determined the atomic structural correlations (Figure 8). The *G*(*r*) peaks (Figure 8) for the average Fe–O distance (vivianite ∼2.21 Å and AFEP ∼2.08 Å) and for Fe–P bonds (vivianite, ∼3.33 Å, and AFEP ∼3.34 Å) were observed.

The Fe–O_PO4_ distances (vivianite at ∼4.08 Å and AFEP at 4.06 Å) were also discernible for both phases. However, the Fe–Fe distance (vivianite at *r* ∼ 4.64, 5.22, and 6.15 Å)^79^ was quite weak or nearly absent for AFEP. The *G*(*r*) decays rapidly with increasing *r* (> 4 Å) for AFEP due to the lack of long-range ordering, indicating the lack of coherence due to its amorphous nature. Contrastingly, the *G*(*r*) of vivianite shows coherent scattering domains to high distances, reflecting its crystalline nature. The results agree with XRD (Figure 1) and TEM-SAED (Figure 2) results described previously.

Contrastingly, the published crystal structure of vivianite^80^ consists of two different octahedrally coordinated Fe^2+^ environments within its lattice, designated as Fe^2+^(A) and Fe^2+^(B). The coordination at the Fe^2+^(A) site shows a tetragonal distortion and near-D_*4h*_ symmetry with four equatorial water ligands at Fe–O distances of 2.213 Å and two axial phosphates at Fe–O distances of 2.030 Å.^80^ The tetragonal compression at this site is evident from the large variation in axial and equatorial Fe–O bond distances.^81^ The near-*C*_*2v*_^82^ symmetry of the Fe^2+^(B) site indicates Fe–O distances of 2.157 and 2.147 Å for bridging and terminal oxygens of the water ligands and 2.101 Å for phosphate oxygens.^80^

The Fe–O bond length variations in vivianite arise due to symmetric effects and variations in orbital hybridizations.^77^ These variations are pronounced in the vivianite pattern (Figure 8) but almost negligible in the AFEP pattern, most likely due to variations in the 4p–3d orbital hybridization of the Fe^2+^ ion. The shorter average Fe–O distances in AFEP were confirmed by both the PDF and EXAFS fitting data, indicating changes in the Fe–O structural geometry that occurred during the transformation. To further contrast the changes in Fe^2+^ 3d electronic transitions, local symmetry, and visible color in the two phases, optical (solid UV–vis to near-IR) absorbance spectra of the AFEP were compared to the literature reference spectrum for naturally occurring vivianite.^77^

Compared to transmission measurements of large (often thin, uniform, and millimeter-sized) natural vivianites, our synthetic vivianite samples, which were micrometer-sized platy florets (Figure F13, Supporting Information), yielded a much weaker signal-to-noise ratio due to the nonuniform path length and partial light scattering. Hence, the literature spectrum for vivianite was used for comparison.

**Figure 9:**
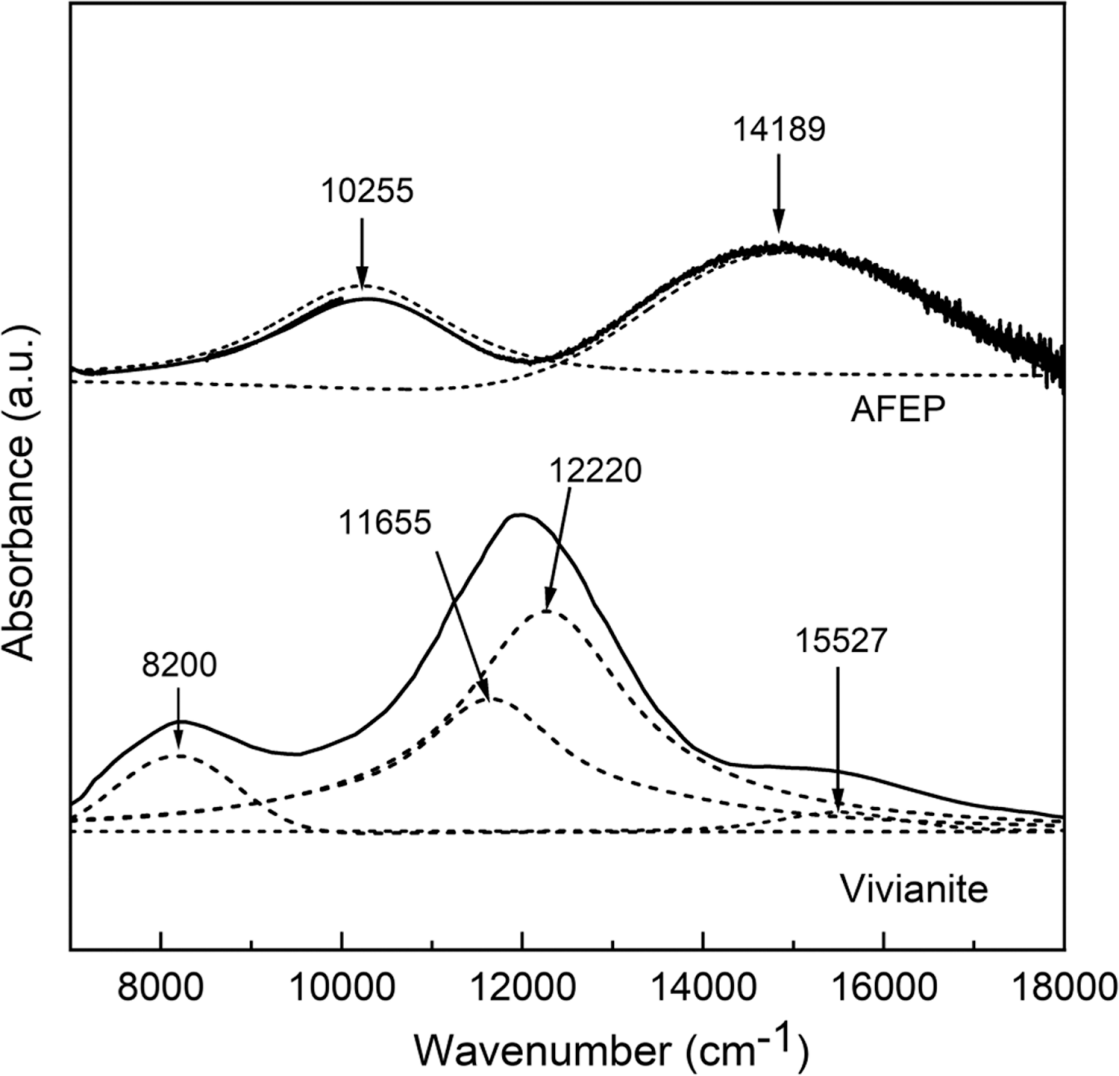
Optical spectra of AFEP and vivianite reference^81^ (solid curves) and deconvoluted pseudo-Voigt component (dotted curves). Arrows mark the optical absorption bands. Noise in the AFEP spectrum at higher wavenumbers is an instrumental artifact.

Nevertheless, electronically, Fe^2+^ in a perfectly octahedral high-spin arrangement (S = 2) has the five 3d orbitals split into three lower-lying *t*_*2g*_-like orbitals (*d*_*x*^2^–*y*^2^_, *d*_*xz*_, and *d*_*yz*_) and two upper-lying *e*_*g*_-like orbitals (*d*_*z*^2^_ and *d*_*xy*_).^81^ The splitting energy between the two *e*_*g*_ levels is directly proportional to the tetragonal distortion in the FeO_6_ octahedron, with a large gap between the two upper-lying *e*_*g*_ orbitals translating to a highly distorted FeO_6_ octahedron. In the optically probed spectral range, the spin-allowed Fe^2+^*d–d* optical transition bands are observed for vivianite at 8200, 11 655, and 12 220 cm^–1^ (Figure 9).^77,81,82^ These are attributed to electronic transitions from the lower-lying *d*_*x*^2^–*y*^2^_ to the upper-lying *e*_*g*_ orbitals.^81^ These multiple bands arise due to octahedrally distorted and structurally nonequivalent Fe^2+^ sites within its lattice.^81,82^ The optical bands at 11 655 and 8200 cm^–1^ arise due to electronic transitions at the Fe^2+^(B) site, showing a slight tetragonal distortion with calculated Fe–O distance variations of ±0.04 Å from the ideal octahedral geometry.^81^ On the contrary, the largely tetragonally distorted Fe^2+^(A) site shows a greater variation of Fe–O distances, reflecting a greater splitting of the two *e*_*g*_-like orbitals. This appears as a strong band at 12 220 cm^–1^ and a theoretically calculated band at ∼4000 cm^–1^.^77,81,82^ In contrast, the experimental optical spectrum of AFEP is characterized by a single band corresponding to the spin-allowed Fe^2+^*d–d* electronic transition at 10 255 cm^–1^ (Figure 9). This single band arises due to electronic excitations from lower-lying *d*_*x*^2^–*y*^2^_ to the two upper-lying degenerate *e*_*g*_ orbitals, indicating a perfectly symmetrical octahedral crystal field. This confirms the presence of a less distorted FeO_6_ octahedron within the AFEP structure, agreeing with the pre-edge XANES fitting results (Figures 5 and F10, Supporting Information).

Since samples were extremely sensitive to Fe oxidation, partial oxidation of the samples was inevitable as the optical measurements were performed outside the anaerobic chamber. Visually, the pristine samples were observed to be pale blue (vivianite) and pale green (AFEP). However, upon aerial exposure, the color of the precipitates darkened due to an increase in the intensity of intravalence charge-transfer (IVCT) bands attributed to Fe^2+^-to-Fe^3+^ electronic transfer at ∼15 000 cm^–1^ in the natural vivianite^82^ and ∼14 000 cm^–1^ in the AFEP pattern (this study). This change reflects and confirms the color perception in the blue and green visible regions, respectively (600–700 nm absorbance).

The XANES, EXAFS, PDF, and optical results together imply that AFEP undergoes significant changes in its local Fe bonding, structure, and symmetry during its transformation to vivianite.

### 3.4 Crystallization Driven by Hydration

**Figure 10:**
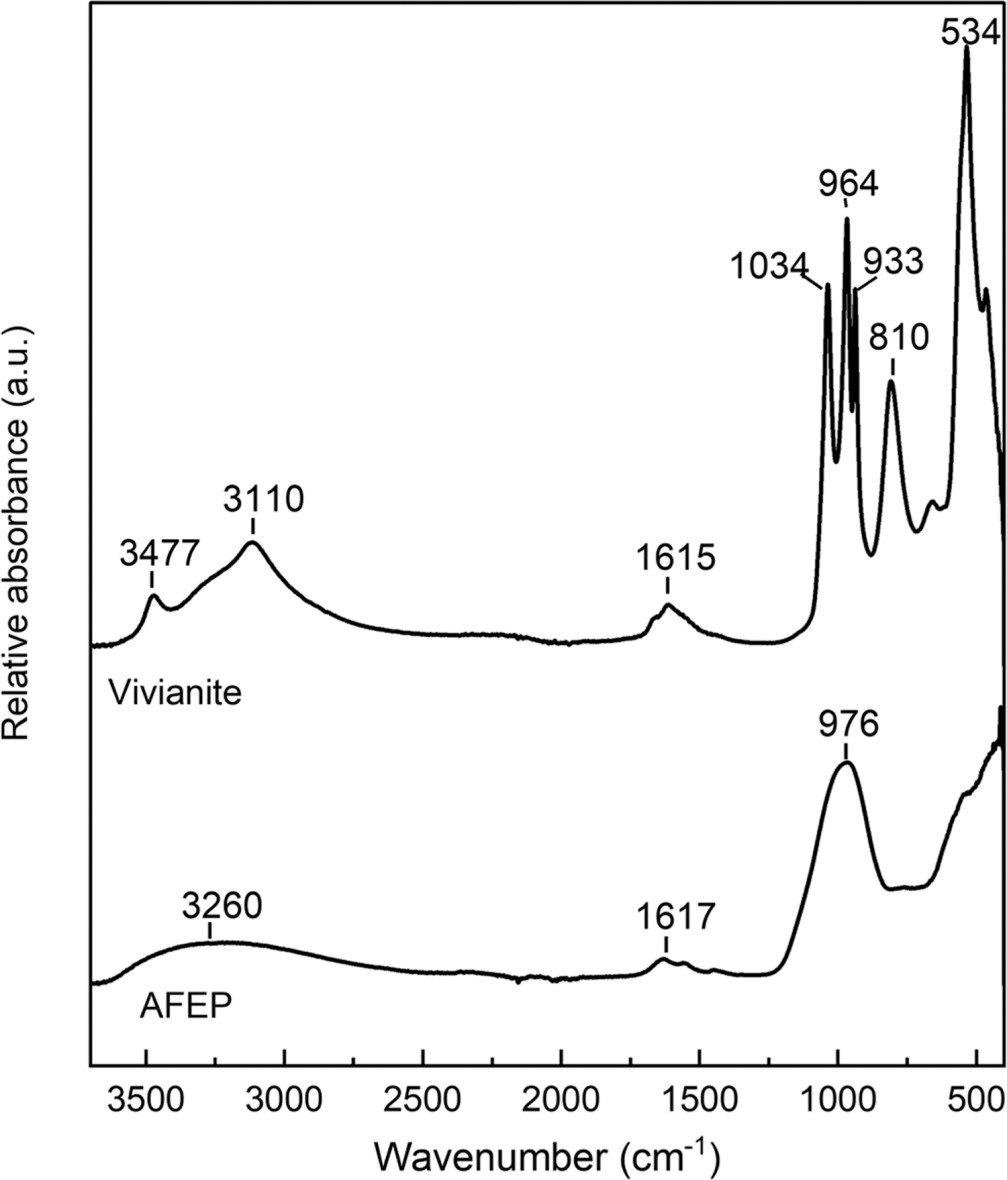
FTIR spectra of AFEP and vivianite with respective characteristic vibrational frequencies marked.

To evaluate the differences in group symmetry and bonding environment of the water and phosphate groups within the two phases, FTIR measurements were performed (Figure 10). The phosphate P–O stretching vibrations ν_1_ (symmetric stretching) and ν_3_ (asymmetric stretching) occur between 1100 and 930 cm^–1^ for the two phases (Figure 10). Our vivianite was characterized by three P–O stretching vibrations at 1034, 964, and 933 cm^–1^, contrary to AFEP, which showed a medium broad peak centered at 976 cm^–1^. The ν_3_ P–O stretching vibrations in AFEP appear merged and therefore difficult to be resolved from one another, producing a single broad peak centered at 976 cm^–1^. In crystalline vivianite, the ν_3_ P–O stretching vibrations are split into three components, which can be explained by lattice constraints,^83^ variable Fe (Fe in two distinct chemical environments),^84^ and subsequently phosphate environments in its crystal structure.

A visually discernible decrease in the splitting of the ν_3_ P–O stretching vibrations has been previously reported for other amorphous metal (Zn, Ca, Pb–NO_3_) phosphate systems.^85−88^ Notably, in the case of lead nitrate phosphate crystals, the increase in ν_3_ splitting in the crystalline phase has been attributed to higher distortions of the PO_4_^3–^ ions compared to those of the isolated PO_4_^3–^ ion due to factor group splitting.^88^

Therefore, changes in splitting features of the P–O stretching vibrations may indicate a decrease in the average tetrahedral distortion^83,88^ in the AFEP, compared to crystalline vivianite, due to a lack of long-range structural arrangement of PO_4_^3–^ units and absence of lattice constraints. The out-of-plane bending mode of the phosphate is visible as a distinct sharp peak at 534 cm^–1^ for vivianite. Furthermore, the O–H stretching vibration appears as a single broad peak centered at ∼3260 cm^–1^ in the AFEP spectrum but it is split into two sharp peaks in vivianite at 3477 and 3110 cm^–1^, reflecting the different water bonding environments in the vivianite crystalline lattice.^84,89^ Similar effects in the O–H stretching vibrational bands have also been reported for amorphous and crystalline Zn-phosphates.^87^

Another notable feature observed in the vivianite IR spectrum is the sharp peak at 810 cm^–1^. This has been reported as a “librational water” stretching mode by Frost et al.,^90^ which arises due to hydrogen-bonded water molecules within its structure.^91^ The librational water bands in IR spectra of crystalline metal hydrates are affected by the H_2_O molecular deformation via H-bonding interactions.^92−94^ Aquo-complexes of transition-metal hydrates exhibit IR-active water librational modes.^93^ The librational IR water band appears distinctly at 810 cm^–1^ in the vivianite spectrum; however, it is not distinguishable for AFEP (Figure 10). The reason may be because it is weak or is overshadowed by the broad P–O stretching peak at 976 cm^–1^. Previous studies have reported that changes in the strength of H-bonding within a crystal lattice causes changes in the intensity and frequency of the librational water bands in their IR spectra.^93^ Therefore, the different intensities of this band in AFEP and vivianite may imply differences in the H-bonding network in the two phases. This may explain the changes in the surrounding local bonding environments, which is corroborated by the lack of long-range ordering as documented previously by its XRD (Figure 1), *G*(*r*) (Figure 8), and SAED (Figure 2C) data. Overall, all the IR data imply that the establishment of a well-defined H-bonding network could accompany this transformation.

The variation in the water contents in our two phases was quantified from the TGA data (Figures F11 and F12, Supporting Information). Calculations based on the net percentage mass loss between 25 and 500°C for repeat experiments yielded an approximate average composition of Fe_3_(PO_4_)_2_·4.75 H_2_O for AFEP. The corresponding TGA data for vivianite (Figure F12, Supporting Information) showed a mass loss of 28.50%, yielding a stoichiometric composition of Fe_3_(PO_4_)_2_·7.98H_2_O. The vivianite water molar ratios well matched the expected stoichiometric composition, i.e., Fe_3_(PO_4_)_2_·8H_2_O. To minimize the contributions from surface-bound water (physisorbed) removal, both the AFEP and crystalline vivianite were dried under vacuum for 6–24 h prior to thermal analysis. The samples showed major water loss from 50 to 110°C (Figures F11 and F12, Supporting Information), and the drying time had no significant effect on the TGA percentage mass loss. Previous TGA–DSC studies of amorphous calcium carbonate (ACC)^22,95^ have shown that ACC dehydrates in a series of steps, with a significant water loss below 115 °C, which has been attributed to the removal of weakly bound or fluid-like mobile as well as restricted H_2_O components from its structure. TGA measurements alone cannot be used to quantify the type of H_2_O components (structural or weakly bound) in a sample because these only detect the rate of mass loss in a sample as a function of temperature, without resolving contributions from individual H_2_O components. However, solid-state ^1^H magic angle spinning (MAS) NMR can be performed for quantification and differentiation of individual proton and H_2_O environments. Nevertheless, vivianite and AFEP have paramagnetic Fe^2+^ in their structures; therefore, their NMR analysis is a challenge and was not performed in this study. Thus, the calculated total water composition of AFEP may include stepwise contributions from both rigidly bound structural and weakly bound H_2_O molecules from AFEP. These data clearly revealed that AFEP has a lower total water content than vivianite, demonstrating that AFEP undergoes a net hydration during its crystallization to vivianite.

This was also supported by the beam-induced damage in the two phases when analyzed by TEM (Figure F14, Supporting Information). During exposure of the vivianite platelets to the focused TEM beam, it was more easily beam-damaged than the aggregated AFEP nanoparticles. Such differences in the reaction to the same beam conditions in materials containing different water contents have been documented before. For example, when observing quartz of similar composition and variable water contents,^96^ the presence of higher amount of water leads to greater beam damage through permanent atomic displacement. Hence, our FTIR and TGA data confirmed the pivotal role of water in the crystallization of vivianite from AFEP. We could document that only structural rearrangement and incorporation of water molecules within the AFEP structure enable its crystallization to vivianite. This conclusion is also supported by the presence of a shorter average Fe–O distance (Figures 6–8; Table T3, Supporting Information) in the AFEP, as compared to that of vivianite. Overall, Fe–O(H_2_O) bonds are slightly elongated as compared to Fe–O(PO_4_) bonds owing to Fe → oxygen → phosphorus π backbonding interactions.^97^ Therefore, a shorter average Fe–O bond length in AFEP is expected due to its lower water content and its higher phosphate mass percentage than vivianite, based on calculated mass percentages from TGA data.

**Figure 11:**
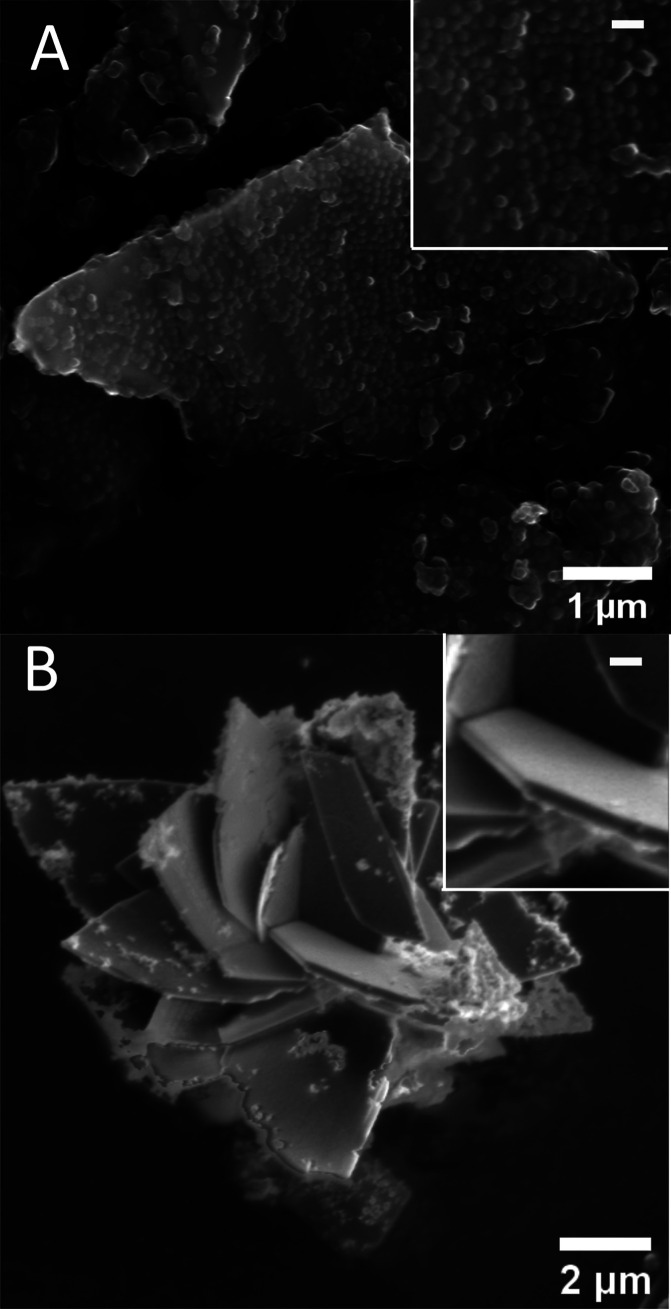
SEM micrographs (A) showing the spherical nanoparticulate texture on the surface of AFEP transformed to vivianite in water after 5 d. (B) Vivianite crystal radial florets showing featureless and smooth surface texture at 1 h (SI 10.19); *insets* at the upper right corners show zooms of the textures, with a white scale bar of 100 nm.

Our results show that an overall hydration of the AFEP nanoparticles occurs upon their transformation to vivianite. To experimentally verify, this dried AFEP powder was suspended in anoxic ultrapure water in a sealed vial and equilibrated inside the anaerobic chamber for up to 5 d. Analysis of the solids after 24 h indicated no changes. However, after 5 d, FTIR analyses of the solids revealed the presence of crystalline vivianite (Figure F15, Supporting Information) based on the strong characteristic phosphate ν_P-O_ (1034, 964, and 933 cm^–1^), ν_O-H_ (3477 and 3110 cm^–1^), δ_H-O-H_ (1615 cm^–1^), and H_2_O libration (810 cm^–1^) peaks. Since the solubility of AFEP is not known, we cannot ascertain its saturation index in this system. A slower rate of its transformation in pure deionized water (>24 h) may be due to supersaturation changes. SEM micrographs of the solids recovered after 5 d (Figures 11A and F15, Supporting Information) revealed large irregular-shaped vivianite crystals with AFEP nanoparticles attached to their surface. In nearly all regions, the formed vivianite showed a surface texture of spherical nanoparticles resembling the starting AFEP uniformly distributed on its surface as shown in Figures 11A and F15 (Supporting Information). The texture of spherical nanoparticles could be distinguished from the loosely attached aggregated AFEP particles based on size, shape, and distribution.

Although the morphology solely cannot be a conclusive proof of the formation mechanism, such nanoparticulate surface textures resembling the dimensions of the initial amorphous intermediates (ACC) are evidence for solid-state transformations in Ca–CO_3_ systems,^4,63,71,72^ wherein crystalline phases exhibit irregular surface textures bearing resemblances to their former nanoparticulate precursors. These differ from the smooth faceted vivianite crystals obtained at a high supersaturation (Figure 11B), which may have formed via ionic attachment from solution^63^ that did not show such a texture within instrumental detection limits, suggesting concurrent pathways of growth and transformation.

Several previous studies reporting the transformation of an amorphous precursor to a crystalline phase in sparingly soluble salt systems most often evidenced a dehydration-induced crystallization pathway. For example, in the Ca–CO_3_ system, the pathway from the hydrated metastable amorphous calcium carbonate (ACC) to thermodynamically more stable crystalline polymorphs such as anhydrous calcite or aragonite proceeds via loss of water.^21,28,70,98−100^ This dehydration step is energetically favorable and exothermic, irreversibly driving the crystallization process.^21^ Similarly, hydrous, amorphous calcium phosphate (ACP) nanoparticles undergo dehydration prior to apatite crystallization.^24,68,101^ On the other hand, in the calcium sulfate system, structural rearrangement, hydration, and particle aggregation of sub-3 nm primary anhydrous but nanocrystalline species is the driving force for gypsum (CaSO_4_·2H_2_O) crystallization.^32^ We clearly document that in the Fe^2+^–PO_4_ system, hydration of the nanoparticulate amorphous precursor phase is the driving force for the crystallization. The incorporation of structural water and rearrangement of parent ions (Fe^2+^ and PO_4_^3–^) favor the transition from dissolved ions to amorphous intermediate to the stable polymorph vivianite. We hence documented that the Fe^2+^–PO_4_ system on the enrichment of water, and not dehydration, leads to the transformation of amorphous intermediates to hydrated crystalline polymorphs. Overall, all of the results presented indicate a lower distortion from ideal octahedral and tetrahedral geometries in the local structure of AFEP, as compared to vivianite. Typically, deviations from regular octahedral geometries, as suggested in the case of vivianite, have kinetic costs due to higher molecular strain (Jahn–Teller distortion).^102^ This factor may kinetically favor the initial formation and stabilization of the less distorted AFEP precursor phase.

## 4 Conclusions

We have shown that vivianite crystallizes via a transient nanoparticulate amorphous Fe^2+^–PO_4_ precursor, AFEP, that precipitates from a solution supersaturated with respect to vivianite. No solubility data for this precursor exist yet, but we have determined its local structure and average composition Fe_3_(PO_4_)_2_·4.75H_2_O and showed that this intermediate is less enriched in water than crystalline vivianite. The water associated with the AFEP nanoparticles structurally rearranges, leading to the formation of a defined H-bonding network, playing a pivotal role in the AFEP transformation to vivianite. Both our *ex* and *in situ* analyses cross-correlated and confirmed that vivianite forms via a predominantly solid-state hydration-driven reaction via AFEP, as opposed to dehydration-driven crystallization at moderately high to low vivianite supersaturations (SI ≤ 10.19).

We suggest that multiple vivianite formation pathways could be present concurrently in a system, as reported for crystallization mechanisms of calcium carbonates^28^ and phosphates.^29,30^ The dominant pathway depends on the systemic vivianite supersaturation. The overall rate of ferrous phosphate nucleation is enhanced, and the induction period decreases with increasing Fe^2+^ and PO_4_^3–^ concentrations. A solid-state transformation via the AFEP is favored at lower supersaturations (SI ≤ 10.19). At very high supersaturations (SI ≥ 12.86), the AFEP most likely dissolves and the growth of vivianite occurs directly from solution via ionic attachment. The AFEP dissolution may be kinetically slow at lower SI. We showed that the lifetime of the AFEP increases at lower vivianite supersaturation and it remains stable once dried and kept anoxically. AFEP also has lower average distortions of its Fe–O octahedron and P–O tetrahedron, as compared to vivianite. This may be linked to its lack of a defined H-bonding network of water molecules and absence of lattice constraints. These factors may be steering this process via the more locally symmetric and kinetically accessible AFEP phase, via the nonclassical route. Synthesis and stabilization of nanoparticulate (high surface area) material such as AFEP may have several potential applications as a cathode material (LiFePO_4_ batteries^45^) or slow-release fertilizer.^5^ Furthermore, the ferrous phosphate system may serve as a model to investigate and draw conclusions for a wider range of hydrated phases with divalent metal ions (having similar hydration enthalpy and ionic radii to Fe^2+^) and phosphates. These insights elucidate the nucleation and growth mechanisms of hydrated crystal phases, and these may be applied to synthesize materials with distinctly different structural and chemical properties, compared to their crystalline counterparts.

## Supporting Information Available

Section S1: preparation of sodium phosphate buffer solution; Section S2: thermodynamic system modeling; Table T1: ICP-OES data for instrumental parameters and quality control (QC) solutions; Figure F1: XRD analysis of crystalline vivianite; Figure F2: STEM–EDS spectrum of precursor at SI 10.19 (30 s); Figure F3: HR-TEM data of precipitates at 100 sec; Figure F4: *ex situ* TEM micrographs of solids at an SI of 7.16: 20 min; Figure F5: STEM–EDS analysis showing aggregated spherical nanoparticles (SI 7.16) at 20 min; Figure F6: XRD pattern and TEM micrographs of solids at SI 7.16 48 h; Section S3 and Figure F7: *in situ* time-resolved pH metric measurements at different supersaturations; Figure F8: XRD pattern of solids at SI 12.86; Figure F9: XANES, EXAFS, FT-EXAFS, and first derivative plots of vivianite and AFEP; Figure F10: pre-edge extraction from Fe K-edge XANES; Table T2: pre-edge analysis and fitting; Table T3: EXAFS modeling and Fe–O/P path fitting; Figure F11: thermogravimetric (TGA) measurement of two AFEP samples; Figure F12: thermogravimetric (TGA) measurement of AFEP and vivianite samples; Figure F13: SEM micrograph of synthetic vivianite at SI 12.86 (20 ± 10 s); Figure F14: TEM images showing variable beam damage in AFEP and vivianite; and Figure F15: FTIR spectrum and SEM micrographs of AFEP after 24 h and 5 d in water (PDF)

## Abbreviations

AFEP: amorphous ferrous phosphate
UV–vis: ultraviolet–visible
ICP-OES: inductively coupled plasma optical emission spectroscopy
SI: saturation index
XRD: X-ray diffraction
PDF: pair distribution function
SEM: scanning electron microscopy
TEM: transmission electron microscopy
SAED: selected area electron diffraction
EDS: energy-dispersive X-ray
FTIR: Fourier transform infrared
TGA: thermogravimetric analysis
XAS: X-ray absorption spectroscopy
XANES: X-ray absorption near-edge structure
EXAFS: extended X-ray absorption fine structure
