# Supplementary material for: Nucleation and Crystallization of Ferrous Phosphate Hydrate via an Amorphous Intermediate
Source: J Am Chem Soc. 2023 Jul 6;145(28):15137–51. doi: 10.1021/jacs.3c01494 (PMC10360157; doi:10.1021/jacs.3c01494)
Supplement: Supplementary file 1 — ja3c01494_si_001.pdf [file ja3c01494_si_001.pdf]

Nucleation and Crystallization of Ferrous Phosphate Hydrate via an Amorphous Intermediate

Alice Paskin,[#,§,*] Thaïs Couasnon,[#] Jeffrey Paulo H. Perez,[#] Sergey S. Lobanov,[#] Roberts Blukis,[#,†] Stefan Reinsch,[‡] Liane G. Benning [#,§]

[#] Deutsches GeoForschungsZentrum, Telegrafenberg, 14473 Potsdam, Germany

[§] Department of Earth Sciences, Free University of Berlin, 12249 Berlin, Germany

[‡] Federal Institute for Materials Research and Testing (BAM), Richard-Willstätter-Straße 11, 12489 Berlin, Germany

[*] Corresponding author

Present Address:

[†] Leibniz-Institut für Kristallzüchtung, Max-Born-Str. 2, 12489 Berlin, Germany

Electronic Supplementary Information (ESI)

Section S1	Preparation of sodium phosphate buffer solution
Section S2	Thermodynamic modeling of the system
Table T1	ICP-OES data for instrumental parameters and quality control (QC) solutions
Figure F1	XRD analysis of crystalline vivianite
Figure F2	STEM-EDS spectrum of precursor at SI 10.19 (30 s)
Figure F3	HR-TEM data of precipitates imaged *ex situ* and at 100 sec
Figure F4	*Ex situ* TEM micrographs of solids from a vivianite precipitation experiment at a SI of 7.16: 20 min
Figure F5	STEM-EDS analysis showing aggregated spherical nanoparticles of iron phosphate (SI 7.16) at 20 min
Figure F6	XRD pattern and TEM micrographs of solids from a vivianite precipitation experiment at SI 7.16 (48 h)
Section S3	pH metric measurements of vivianite precipitation at different supersaturations

Figure F7 — *In situ* time-resolved pH metric measurements of vivianite precipitation at different supersaturations at pH 7.2.

Figure F8 — XRD pattern of solids from a vivianite precipitation experiment at SI 12.86

Figure F9 — XANES, EXAFS, FT-EXAFS and first derivative XANES plots of vivianite and AFEP

Figure F10 — Pre-edge extraction from Fe-K edge XANES spectrum of AFEP

Table T2 — Pre-edge analysis and fitting for AFEP

Table T3 — EXAFS modeling and Fe-O/P path fitting

Figure F11 — Thermogravimetric (TGA) measurement and the first derivative of the TGA plot (dTG) of two AFEP samples under Ar gas flow

Figure F12 — Thermogravimetric (TGA) measurement of AFEP and vivianite samples under Ar gas flow

Figure F13 — SEM micrograph of synthetic vivianite collected from an experiment at SI 12.86 (20 ± 10 s)

Figure F14 — TEM images showing variable beam damages in AFEP and vivianite

Figure F15 — FTIR spectrum of AFEP after 24 h and 5 d in water; SEM micrographs of solids after suspending AFEP for 5 d in anoxic DI water

Section S1. Preparation of sodium phosphate buffer solution

Preparation of sodium phosphate buffer solution was performed by mixing calculated amounts of NaH_2PO_4 and Na_2HPO_4 solids in the required volume of water to achieve a particular pH and concentration of phosphate in solution. The calculations were based on Henderson Hasselbalch equation taking the second dissociation constant of phosphoric acid as $K_2 = 6.2 \cdot 10^{-8}$

$$K_2 = \frac{[H^+][HPO_4^{2-}]}{[H_2PO_4^-]}$$

The calculated $pK_2 = 7.21$ for the chosen range for vivianite precipitation (pH 6 – 8). Hence, the following equation can be used to calculate the amount of di- and mono-basic phosphates to prepare the buffer solution of required pH

$$X_{NaH_2PO_4} = \frac{X_{PO_4}}{10^{(pH-7.21)} + 1}$$

Where $X_{NaH_2PO_4}$ is the calculated concentration with the input parameters of required final phosphate concentration (X_{PO_4}) and pH. Substituting the calculated amount of $X_{NaH_2PO_4}$ from the above equation, the amount of $X_{Na_2HPO_4}$ can be calculated as

$$X_{Na_2HPO_4} = X_{PO_4} - X_{NaH_2PO_4}$$

Therefore, to prepare 250 mL of 10 mM phosphate buffer of pH 7.2, the di-basic phosphate reagent Na_2HPO_4 (Sigma Aldrich, 99.98 %, anhydrous; 150.1 mg) and monobasic NaH_2PO_4 powder (Alfa Aesar, 99.8 %; 175 mg) were weighed and transferred to the anaerobic chamber in a 250 mL volumetric flask and degassed O_2-free Milli-Q water (18.2 MΩ·cm) was added to it, the flask was closed and shaken to dissolve the salts to obtain a homogeneous solution. The phosphate buffer solution was stored inside the anaerobic chamber inside an acid cleaned and rinsed polypropylene (PP) bottle until further use.

Section S2. Thermodynamic modeling of the system

Thermodynamic modeling of the system based on calculations on PHREEQC software (Version 3) [1] using the Thermoddem database [2]

The solubility of vivianite in water can be expressed by the following chemical equilibrium:

$$Fe_3(PO_4)_2 \cdot 8H_2O + 2\,H^+ \rightarrow 3\,Fe^{2+} + 2\,HPO_4^{2-} + 8\,H_2O$$

The solubility product of vivianite is $10^{-35.767}$ and its saturation index (SI) is defined as [3]:

$$SI_{Vivianite} = log\frac{[a_{Fe^{2+}}]^3 \cdot [a_{HPO4^{2-}}]^2}{[H^+]^2 \cdot K_{sp(vivianite)}}$$

At pH 7.2 and a temperature of 25˚C, p_e of -1 (anoxic system) and 1 atm pressure, the system composition was input as:

Species	SI 1 (10^{-3} mol L^{-1})	SI 2 (10^{-3} mol L^{-1})	SI 3 (10^{-3} mol L^{-1})
Fe^{2+}	5	0.5	50
HPO_4^{2-}	5	0.5	50
NH_4^+	10	1	100
SO_4^{2-}	10	1	100
Na^+	5	0.5	50

The following were the calculated solid SI at different concentrations:

Phase	SI 1	SI 2	SI 3
Melanterite ($FeSO_4 \cdot 7H_2O$)	-3.22	-4.55	-2.16
Vivianite	10.19	7.16	12.86

Table T1. ICP-OES data for instrumental parameters and quality control (QC) solutions

ICP-OES data for quality control (QC) solutions prepared from purely elemental standards (Merck, CentiPur). The mean results of n replicate analyses are given with standard deviation (SD) and relative standard deviations (RSD)

**Parameters**	**Fe (mg L^{-1})**	**P (mg L^{-1})**
Wavelength	261.382 nm	213.618 nm
Instrumental limits		
Limit of detection (LoD)	$0.014 \cdot 10^{-3}$	0.0040
Limit of quantification (LoQ)	0.002	0.013
Quality Control (QC)		
QC verify (n = 5)	0.406	0.205
SD	0.008	0.005
RSD	2.0%	2.5%
2RSD	4.1%	5.1%
Reference value	0.406	0.205
Deviation from reference value	0.01%	0.05%

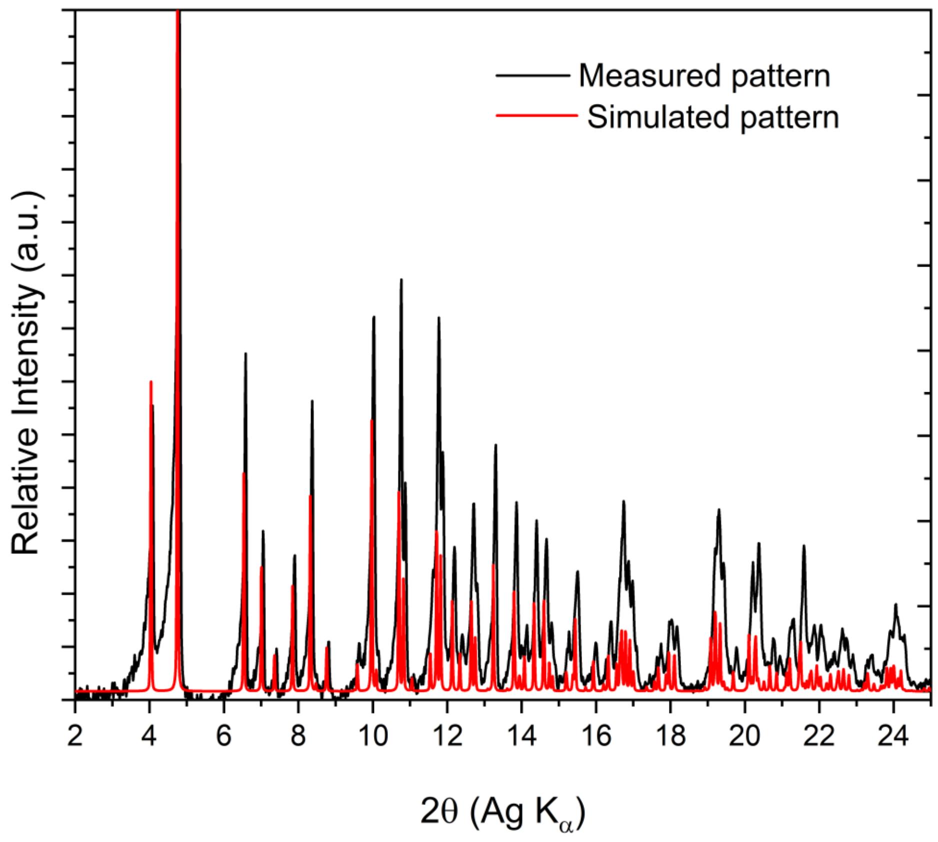

Figure F1. Powder X-ray diffraction (XRD) pattern of the collected precipitates (black) and calculated XRD pattern for crystalline vivianite [4] (red). SI 10.19 (1 h)

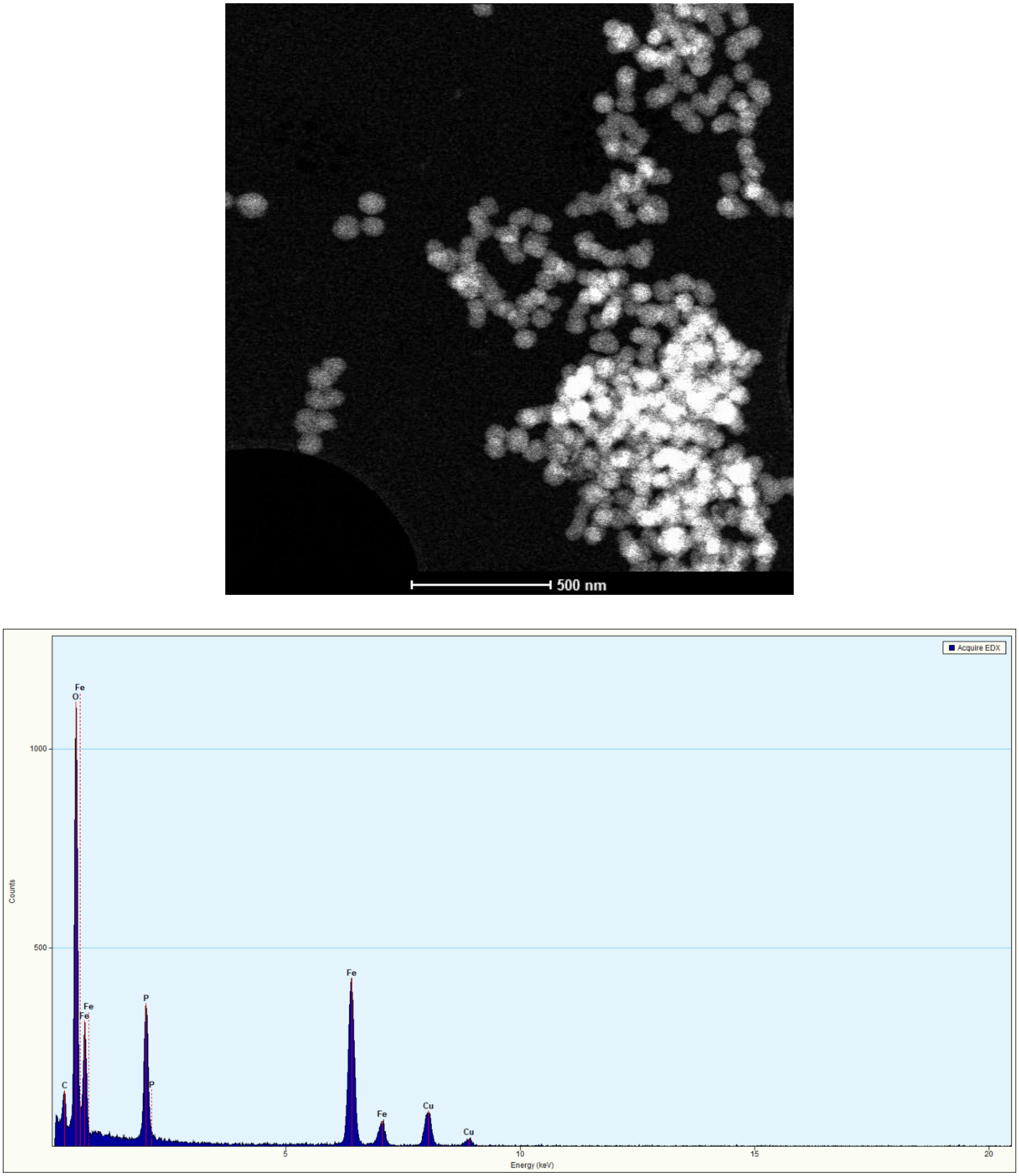

Figure F2. STEM-EDS analysis of precursor indicating an iron phosphate phase (SI 10.19), the Cu signal comes from the copper grid background. SI 10.19 – 30 s.

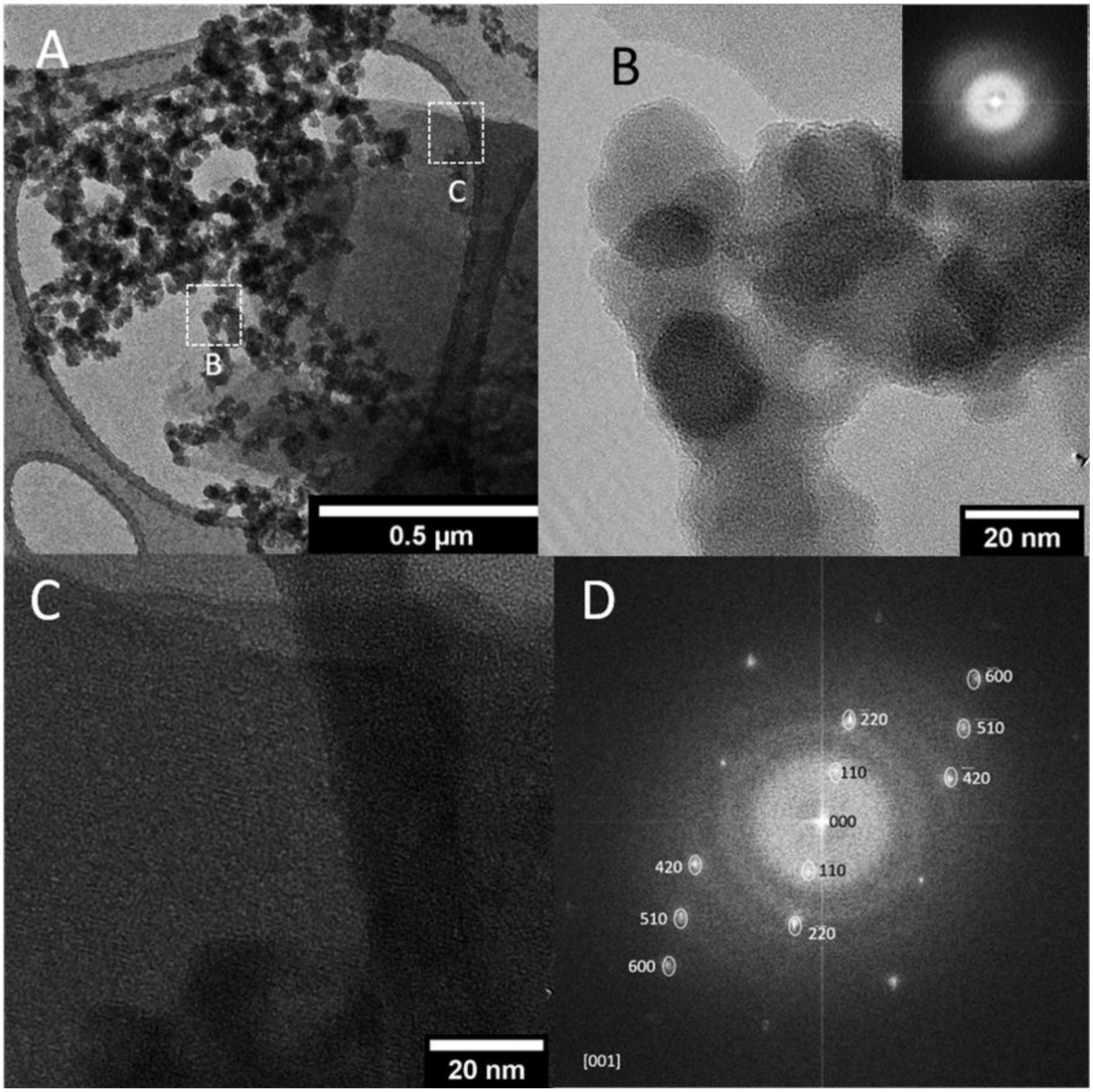

Figure F3. (A) TEM micrograph of solids obtained from batch reaction at 100 s with squared areas representing the locations of HRTEM analysis for B and C; (B) HR-TEM of area B showing amorphous intermediate (*inset* FFT pattern); (C) HR-TEM of area C with corresponding crystalline FFT pattern for vivianite indexed in (D). SI 10.19.

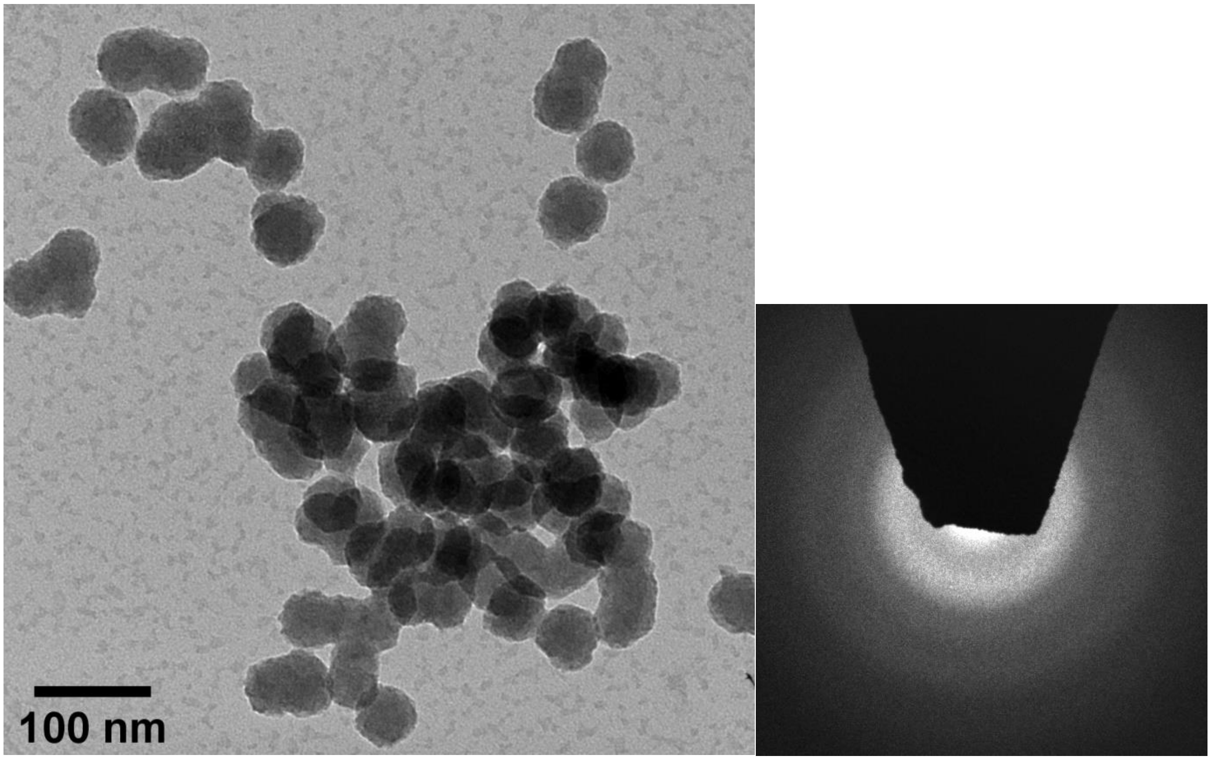

Figure F4. *Ex situ* TEM micrographs of solids from a vivianite precipitation experiment at a SI of 7.16: 20 min showing spherical aggregated nanoparticles (~ 50 nm in diameter) and corresponding SAED pattern showing diffuse rings characteristic of an amorphous material.

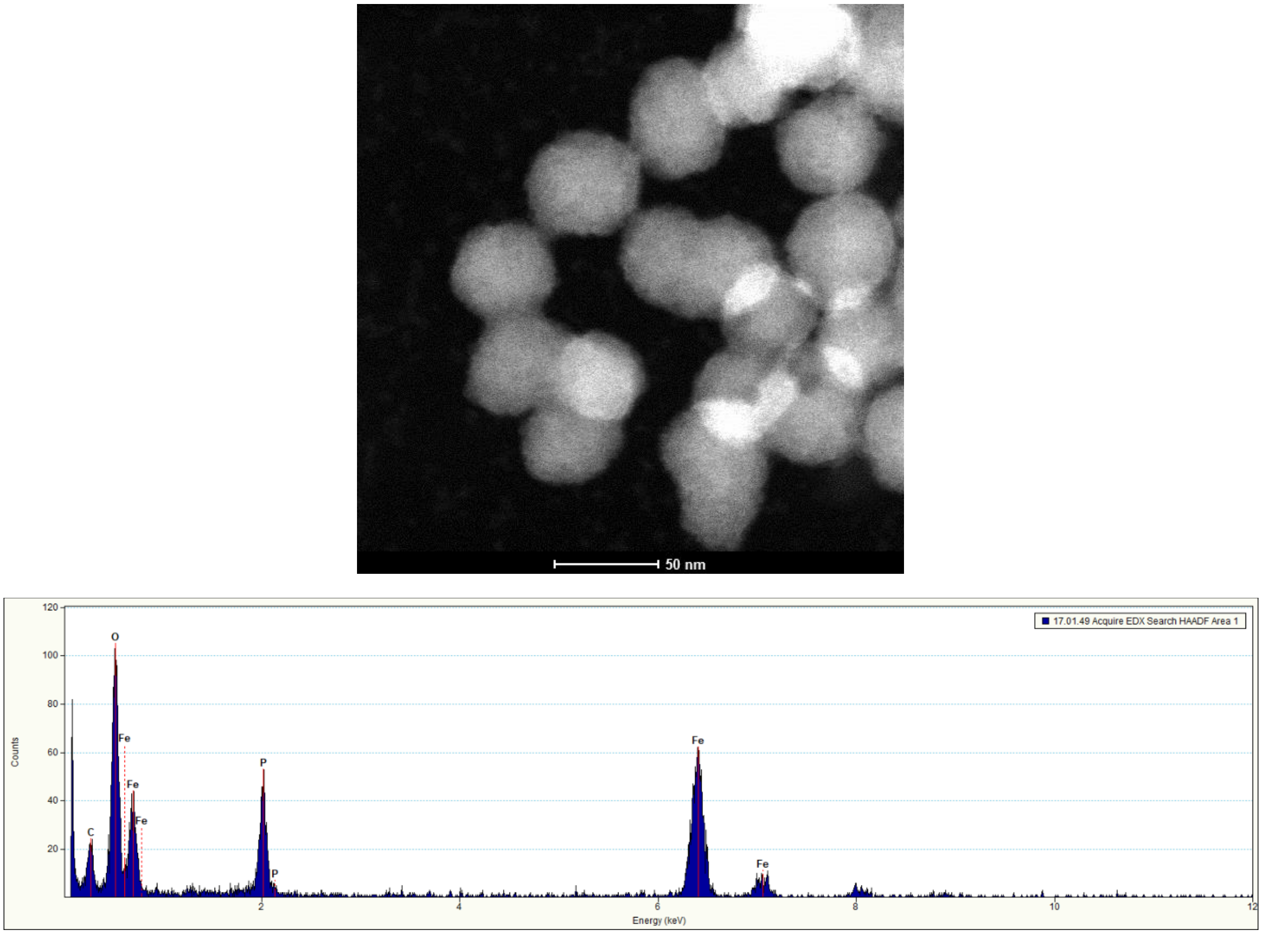

Figure F5. STEM-EDS analysis showing aggregated spherical nanoparticles of iron phosphate (SI 7.16) at 20 min.

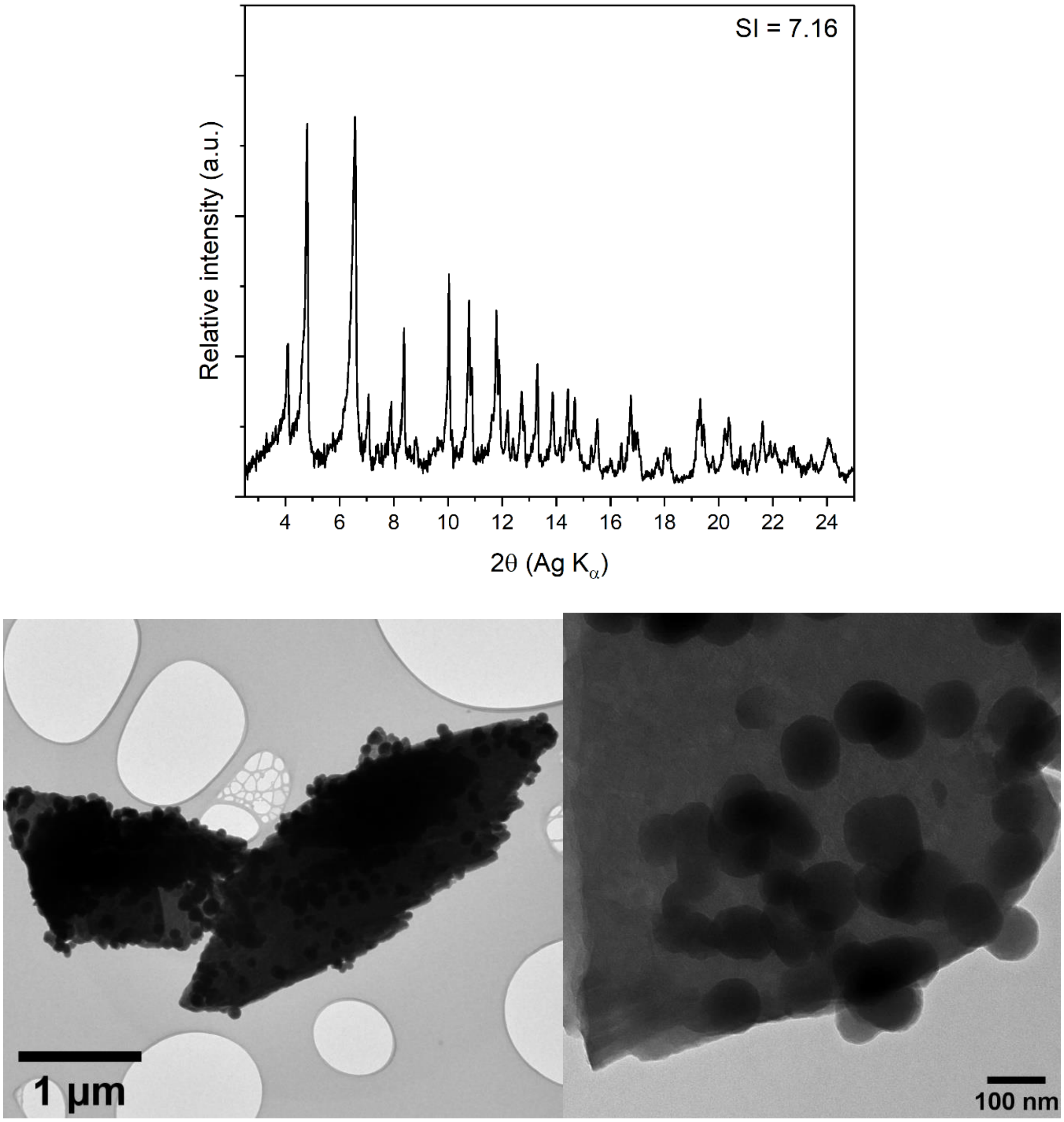

Figure F6. XRD pattern of solids from a vivianite precipitation experiment at SI 7.16 48 h after filter quenching (*above*). TEM micrographs of the solids from the reaction mixture at 48 h showing vivianite crystals with aggregated amorphous precursor on its surface (*below*).

Section S3: *In situ* pH metric measurements of vivianite precipitation at different supersaturations

Due to the protonated speciation of the phosphate (HPO_4^{2-} and $H_2PO_4^{-}$) at pH 6.5 – 8.0 [5], the precipitation of vivianite near neutral pH leads to a release of protons and causes a decrease in the total solution pH [6]. The rate of the initial decrease can reflect the rate of initial nucleation of ferrous phosphate phases. At higher supersaturation (SI 12.86) nucleation was almost instantaneous with an induction period of ~ 5 s, observed as a sudden and steep decrease in pH curve. At lower supersaturation (SI 7.16) there was an increase in the induction period (~ 12 s) , as expected [7]. The fast-initial decrease at each supersaturation was proportional to the amount of ferrous phosphate precipitated (H^+ released) in solution.

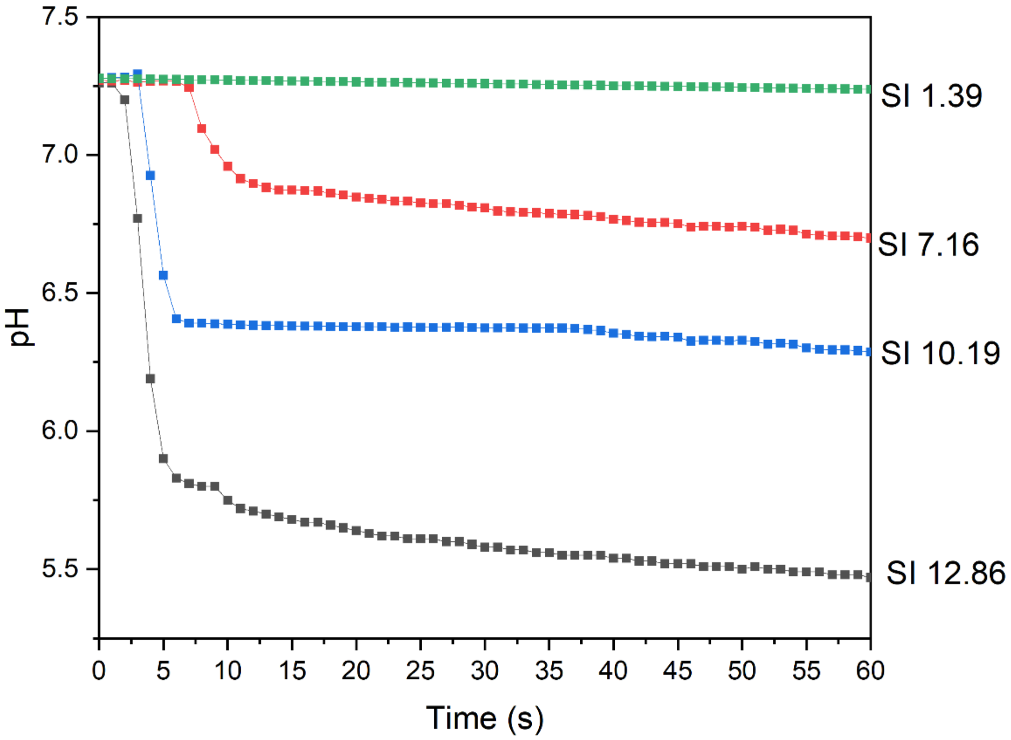

Figure F7. In situ time resolved pH metric measurements of vivianite precipitation at different supersaturations at pH 7.2.

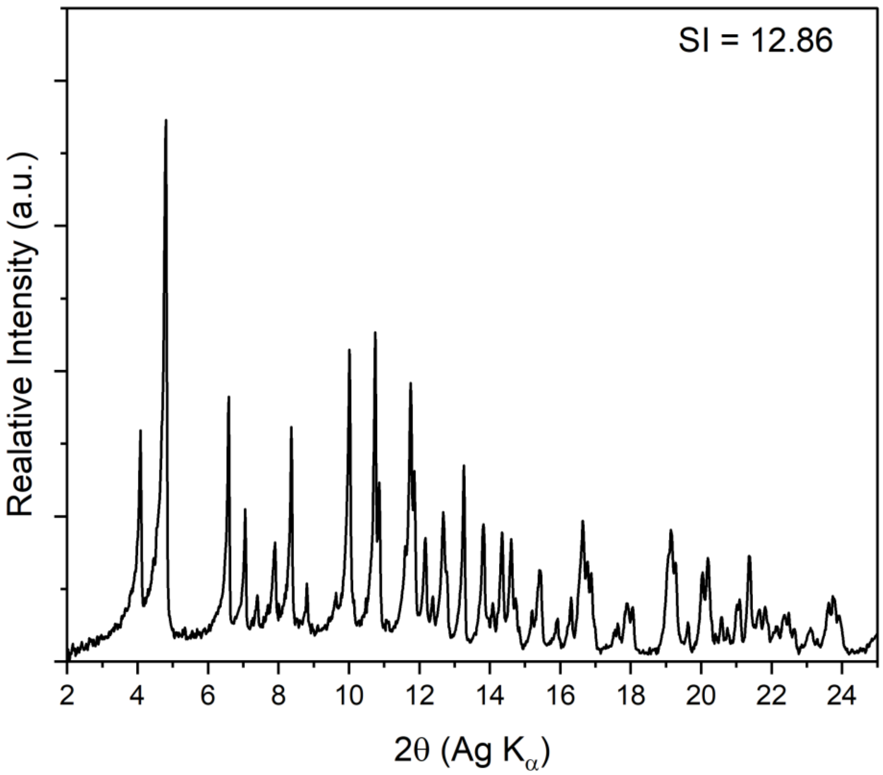

Figure F8. XRD pattern of solids from a vivianite precipitation experiment at SI 12.86 immediately after filter quenching, time taken for filtration ~ 20 s.

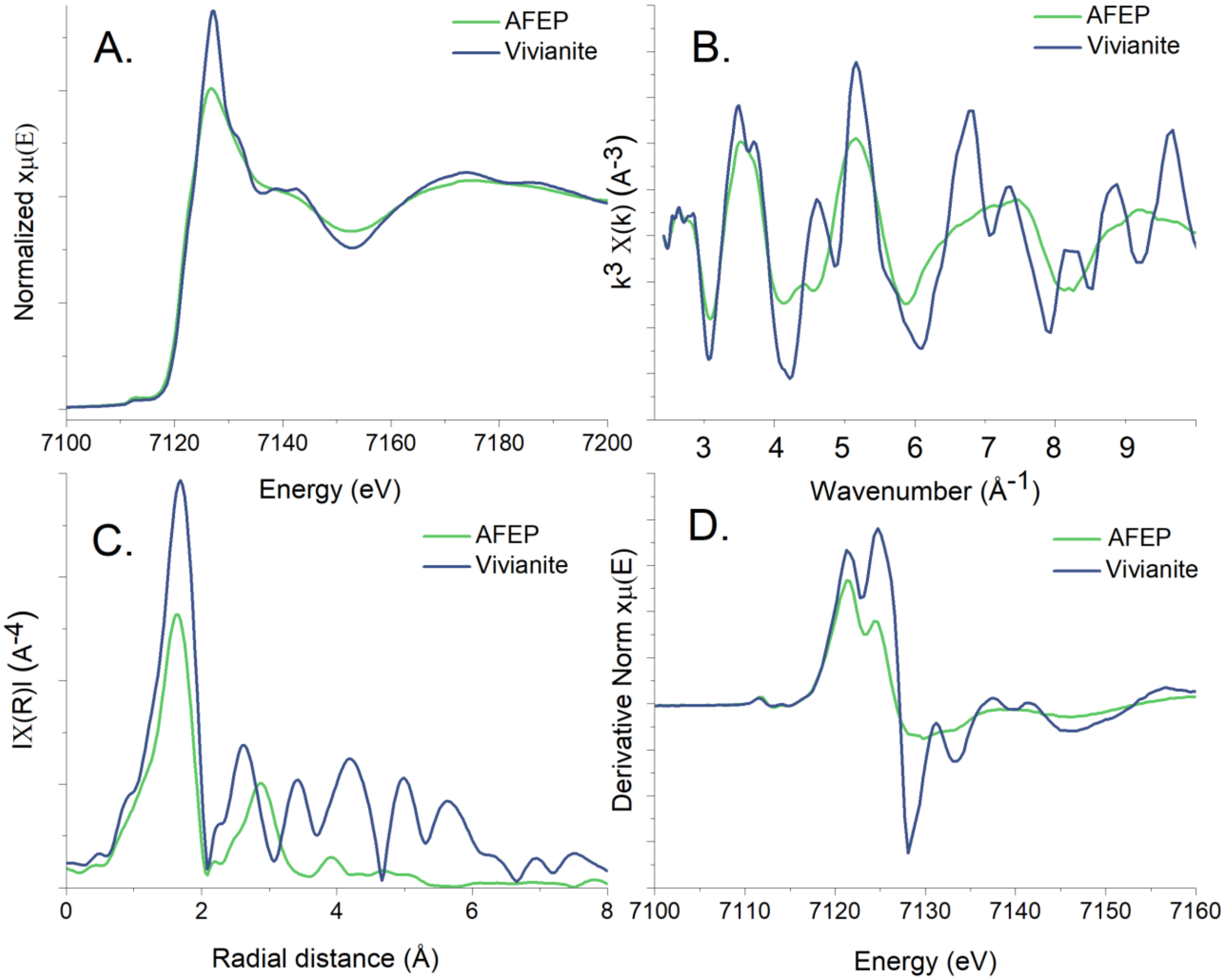

Figure F9. (A) Normalized Fe K-edge XANES; (B) k^3-weighted $\chi(k)$ EXAFS spectra; (C) Radial distribution function via FT-EXAFS spectra; (D) First derivative of $\chi(k)$; for AFEP and vivianite respectively.

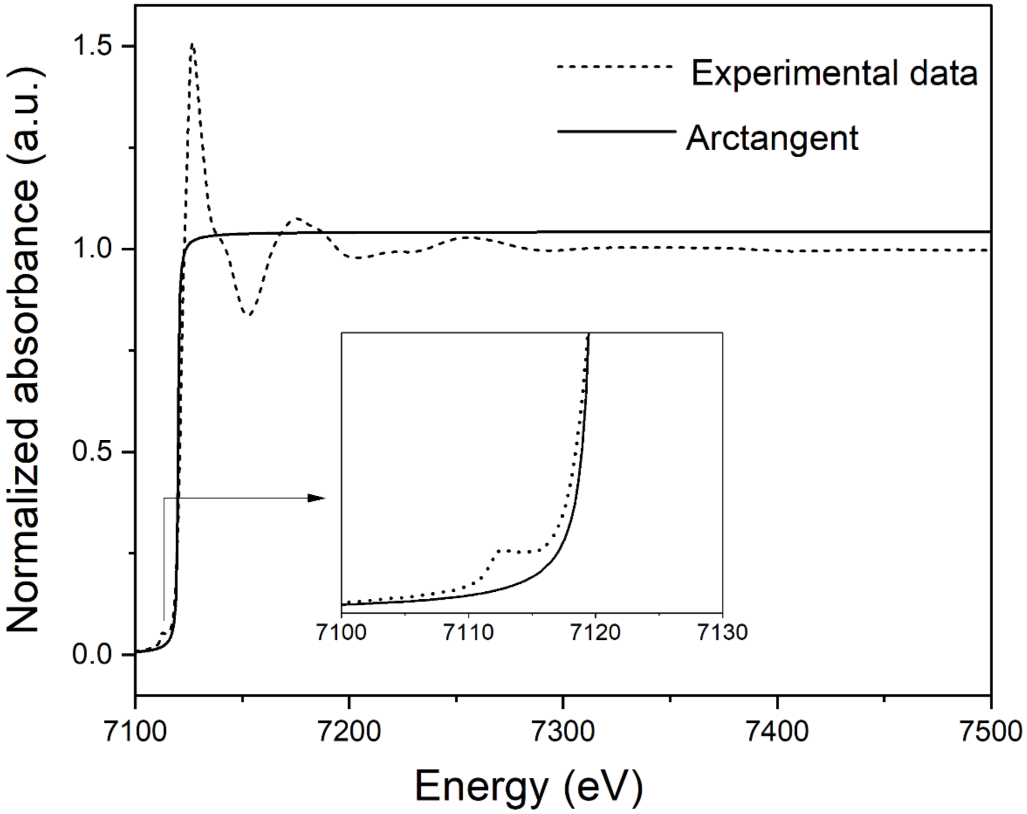

Figure F10. Pre-edge extraction [8] from Fe-K edge XANES spectrum of AFEP via subtraction of a modeled arctangent function

Table T2.

Fe-K edge XANES pre-edge fitting results for AFEP via Fityk software [9] for Pseudo-Voigt fits (50:50) and Levenberg-Marqurадt (least squares) method.

Component type	Component position (eV)	Height	Area [b]	WSSR [c]
Peak 1	7112.70	0.01811	0.0416	-
Peak 2	7112.24	0.00445	0.0056	-
Peak 3	7114.44	0.00703	0.0110	-
Cumulative peak fit [a]	7112.95	-	0.0578 [d]	0.98809

[a] 'Cumulative peak fit' corresponds to the centroid position

[b] Integrated area under the peak (OriginPro (2021) software (OriginLab Corporation, Northampton, MA, USA)

[c] WSSR denotes weighted sum of squared residuals

[d] Represents total area of the cumulative peak fit

Table T3. EXAFS analysis via shell-fit results obtained by fitting Fe-K edge EAXFS of synthesized AFEP sample

Path	CN[a]	R (Å)[b]	σ^2 (Å^2)[c]	ΔE_0 (eV)[d]	Red. χ^2 [e]	R-factor
Fe-O	5.4 (0.5)	2.10 (0.01)	0.010 (0.001)	3.4 (0.9)	21.2	0.027
Fe-P	3.5 (1.0)	3.34 (0.01)	0.011 (0.003)			

Best fit value reported with uncertainty in statistical fit for the last digit as shown in parenthesis.

[a] Coordination numbers

[b] Mean half-path length

[c] Debye-Waller factor

[d] Energy-shift parameter

[e] Reduced χ^2 as using the SIXPack interface to the IFEFFIT XAFS analysis package [10]

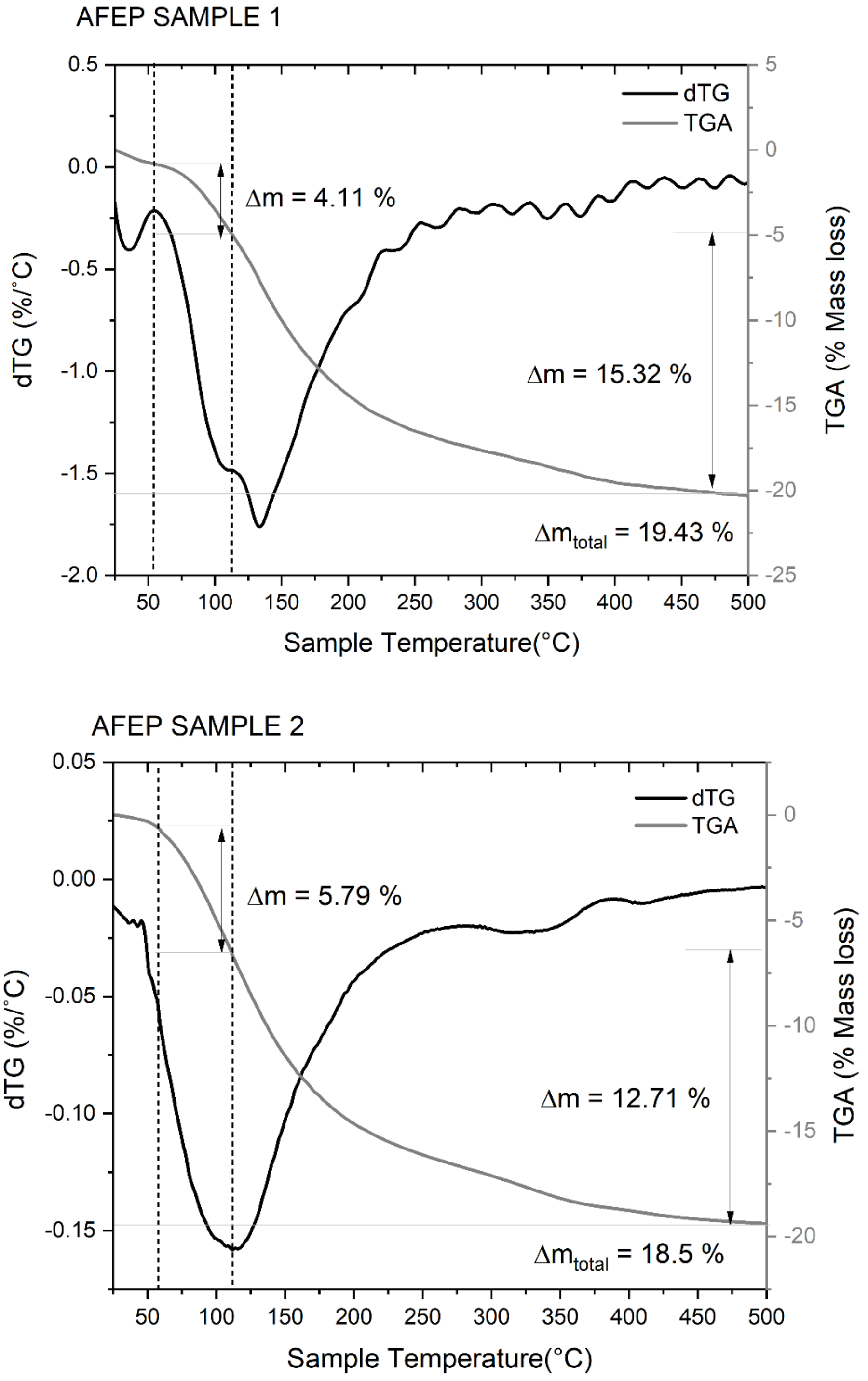

Figure F11. Thermogravimetric (TGA) measurement and the first derivative of the TGA plot (dTG) of two AFEP samples under Ar gas flow. Sample 1 was dried under vacuum for 6 h and sample 2 was dried for 24 h, prior to analysis.

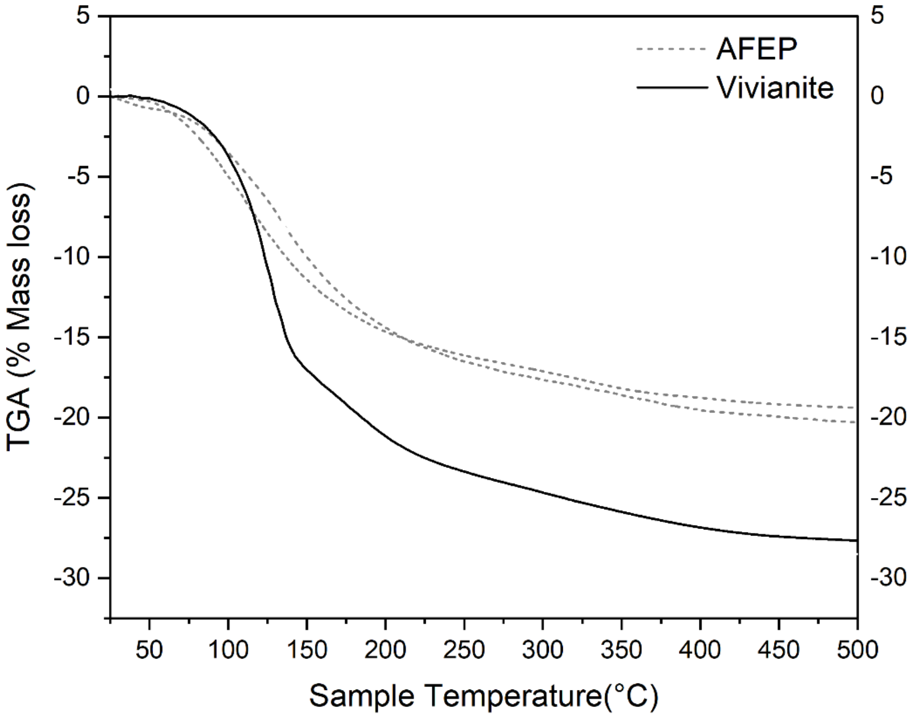

Figure F12. Thermogravimetric (TGA) measurement of AFEP (repeat) and vivianite samples under Ar gas flow.

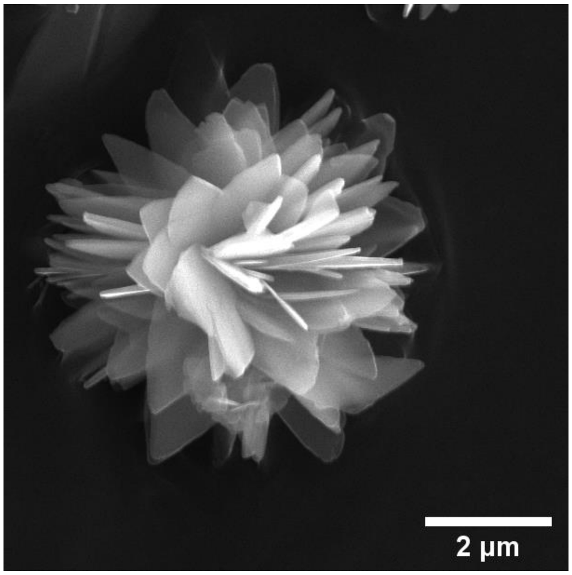

Figure F13. SEM micrograph of synthetic vivianite collected from an experiment at SI 12.86 (20 ± 10 s) showing micrometer ranged platy rosette

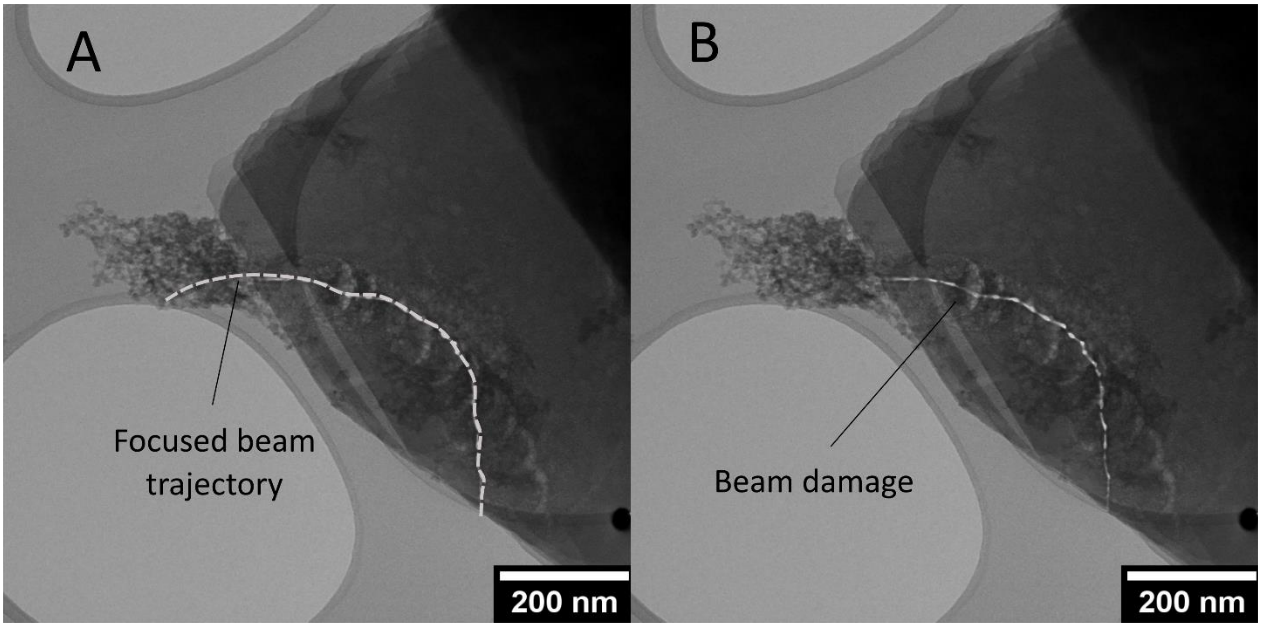

Figure F14. (A) TEM micrograph of aggregated AFEP nanoparticles and crystalline vivianite showing the manually focused electron beam trajectory represented by a white dashed line (dose rate 1170 e Å^{-2} s^{-1}). **(B)** TEM micrograph showing the observed beam damage, indicating greater beam damage for crystalline vivianite as compared to AFEP nanoparticles. SI 10.19.

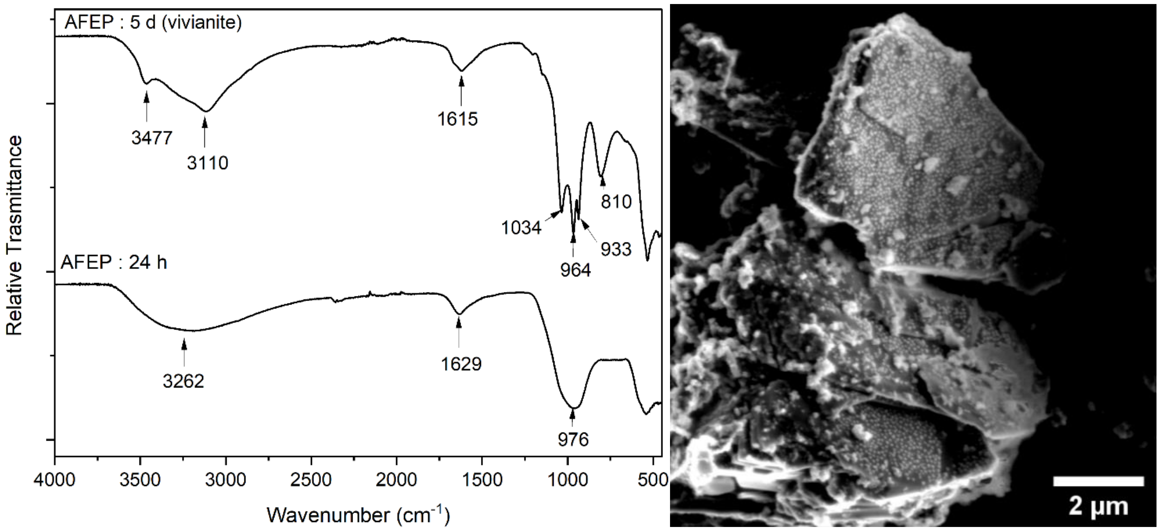

Figure F15. **(Supporting information)** FTIR spectra of dried solid obtained after suspending AFEP (10 mg) in 50 ml anoxic deionized water in crimped vials inside the anaerobic chamber after 24 h and 5 d. Sample at 24 h showed no observable changes but presence of vivianite was observed after 5 days (*left*), SEM micrographs of solids after suspending AFEP for 5 d in anoxic DI water showing crystalline vivianite with nanoparticulate texture on their surface (*right*).

References

1. Parkhurst, D. L., C.A.J. Appelo, *Guide to PHREEQC : a computer program for speciation, reaction-path, advective-transport, and inverse geochemical calculations*. Lakewood, Colo. : U.S. Dept. of the Interior, U.S. Geological Survey ; Denver, CO : Earth Science Information Center, Open-File Reports Section [distributor], 1995.: 1995.
2. Blanc, P.; Lassin, A.; Piantone, P.; Azaroual, M.; Jacquemet, N.; Fabbri, A.; Gaucher, E. C., Thermoddem: A geochemical database focused on low temperature water/rock interactions and waste materials. *Applied Geochemistry* **2012,** *27* (10), 2107-2116.
3. Alborno, A.; Tomson, M. B., The temperature dependence of the solubility product constant of vivianite. *Geochimica Et Cosmochimica Acta* **1994,** *58* (24), 5373-5378.
4. Capitelli, F.; Chita, G.; Ghiara, M. R.; Rossi, M., Crystal-chemical investigation of vivianite minerals. *Zeitschrift Fur Kristallographie-Crystalline Materials* **2012,** *227* (2), 92-101.
5. Nriagu, J. O., Stability of vivianite and ion-pair formation *Geochimica et Cosmochimica Acta* **1972,** *36* (4), 459-470.
6. Goedhart, R.; Müller, S.; van Loosdrecht, M. C. M.; van Halem, D., Vivianite precipitation for iron recovery from anaerobic groundwater. *Water Research* **2022,** *217*, 118345.
7. Nielsen, A. E., Nucleation and Growth of Crystals at High Supersaturation. *Kristall und Technik* **1969,** *4* (1), 17-38.
8. Wilke, M.; Farges, F.; Petit, P. E.; Brown, G. E.; Martin, F., Oxidation state and coordination of Fe in minerals: An FeK-XANES spectroscopic study. *American Mineralogist* **2001,** *86* (5-6), 714-730.
9. Wojdyr, M., Fityk: a general-purpose peak fitting program. *Journal of Applied Crystallography* **2010,** *43*, 1126-1128.
10. Webb, S. M., SIXpack: a graphical user interface for XAS analysis using IFEFFIT. *Physica Scripta* **2005,** *T115*, 1011-1014.
